# Proteomic analysis of ferroptosis pathways reveals a role of CEPT1 in suppressing ferroptosis

**DOI:** 10.1093/procel/pwae004

**Published:** 2024-03-02

**Authors:** Xiaoguang Liu, Zhen Chen, Yuelong Yan, Fereshteh Zandkarimi, Litong Nie, Qidong Li, Amber Horbath, Kellen Olszewski, Lavanya Kondiparthi, Chao Mao, Hyemin Lee, Li Zhuang, Masha Poyurovsky, Brent R Stockwell, Junjie Chen, Boyi Gan

**Affiliations:** Department of Experimental Radiation Oncology, The University of Texas MD Anderson Cancer Center, Houston, TX 77030, USA; Department of Experimental Radiation Oncology, The University of Texas MD Anderson Cancer Center, Houston, TX 77030, USA; Department of Experimental Radiation Oncology, The University of Texas MD Anderson Cancer Center, Houston, TX 77030, USA; Department of Biological Sciences and Department of Chemistry, Columbia University, New York, NY 10027, USA; Department of Experimental Radiation Oncology, The University of Texas MD Anderson Cancer Center, Houston, TX 77030, USA; Department of Experimental Radiation Oncology, The University of Texas MD Anderson Cancer Center, Houston, TX 77030, USA; Department of Experimental Radiation Oncology, The University of Texas MD Anderson Cancer Center, Houston, TX 77030, USA; Kadmon Corporation, LLC (A Sanofi Company), New York, NY 10016, USA; The Barer Institute, Philadelphia, PA 19104, USA; Kadmon Corporation, LLC (A Sanofi Company), New York, NY 10016, USA; Department of Experimental Radiation Oncology, The University of Texas MD Anderson Cancer Center, Houston, TX 77030, USA; Department of Experimental Radiation Oncology, The University of Texas MD Anderson Cancer Center, Houston, TX 77030, USA; Department of Experimental Radiation Oncology, The University of Texas MD Anderson Cancer Center, Houston, TX 77030, USA; Kadmon Corporation, LLC (A Sanofi Company), New York, NY 10016, USA; Department of Biological Sciences and Department of Chemistry, Columbia University, New York, NY 10027, USA; Department of Experimental Radiation Oncology, The University of Texas MD Anderson Cancer Center, Houston, TX 77030, USA; The University of Texas MD Anderson Cancer Center UTHealth Graduate School of Biomedical Sciences, Houston, TX 77030, USA; Department of Experimental Radiation Oncology, The University of Texas MD Anderson Cancer Center, Houston, TX 77030, USA; The University of Texas MD Anderson Cancer Center UTHealth Graduate School of Biomedical Sciences, Houston, TX 77030, USA

**Keywords:** proteomics, ferroptosis, CEPT1, LPCAT3

## Abstract

Ferroptosis has been recognized as a unique cell death modality driven by excessive lipid peroxidation and unbalanced cellular metabolism. In this study, we established a protein interaction landscape for ferroptosis pathways through proteomic analyses, and identified choline/ethanolamine phosphotransferase 1 (CEPT1) as a lysophosphatidylcholine acyltransferase 3 (LPCAT3)-interacting protein that regulates LPCAT3 protein stability. In contrast to its known role in promoting phospholipid synthesis, we showed that CEPT1 suppresses ferroptosis potentially by interacting with phospholipases and breaking down certain pro-ferroptotic polyunsaturated fatty acid (PUFA)-containing phospholipids. Together, our study reveals a previously unrecognized role of CEPT1 in suppressing ferroptosis.

## Introduction

Ferroptosis represents a unique form of regulated cell death driven by detrimental lipid peroxidation in an iron-dependent manner (Dixon et al., 2012). Accordingly, ferroptosis is regulated by diverse proteins involved in lipid metabolism and peroxidation, iron metabolism, and antioxidant defense (Jiang et al., 2021; Stockwell, 2022). Specifically, due to the bis-allylic structure in polyunsaturated fatty acids (PUFAs; fatty acids with more than one double bond), PUFA-containing phospholipids (PUFA-PLs) are susceptible to lipid peroxidation, which when accumulated to lethal levels can damage membrane integrity and induce ferroptotic cell death (Jiang et al., 2021; Stockwell, 2022). PUFA-PL synthesis is mediated by lipid metabolism enzymes such as long-chain fatty-acid-CoA ligase 4 (ACSL4) and lysophosphatidylcholine acyltransferase 3 (LPCAT3), and PUFA-PL peroxidation involves non-enzymatic Fenton reaction and other proteins that participate in lipid peroxidation, such as cytochrome P450 oxidoreductase (POR) and arachidonate lipoxygenase (ALOX) 12 and 15. Inactivation of these proteins involved in PUFA-PL synthesis and peroxidation suppresses ferroptosis (Dixon et al., 2015; Doll et al., 2017; Wenzel et al., 2017; Yan et al., 2021; Yang et al., 2016; Yuan et al., 2016; Zou et al., 2020).

Iron participates in lipid peroxidation by both promoting Fenton reaction and serving as the co-factor for POR and ALOX12/15 (Chen et al., 2020a). Correspondingly, proteins involved in iron transport, storage, metabolism, and export regulate ferroptosis by affecting intracellular labile iron pools. For example, nuclear receptor coactivator 4 (NCOA4)-mediated ferritinophagy promotes ferroptosis by increasing the level of the labile iron pool (Gao et al., 2016; Hou et al., 2016).

Cells have evolved at least four ferroptosis defense systems to counteract adverse effects caused by lipid peroxidation and to maintain cell homeostasis (Lei et al., 2022). The major ferroptosis defense mechanism involves the solute carrier family 7 member 11 (SLC7A11; also called xCT), glutathione (GSH), and glutathione peroxidase 4 (GPX4) in the SLC7A11-GSH-GPX4 system, in which cystine imported by SLC7A11 is reduced to cysteine, which provides the rate-limiting precursor for synthesizing GSH via glutamate-cysteine ligase (GCL, which consists of GCLC and GCLM subunits) and glutathione synthetase (GSS) (Koppula et al., 2018). GSH is then used as the co-factor by GPX4 to quench lipid peroxides and suppress ferroptosis (Friedmann Angeli et al., 2014; Yang et al., 2014). Other proteins involved in GPX4-independent ferroptosis defense pathways include ferroptosis suppressor protein 1 (FSP1; also called AIFM2), dihydroorotate dehydrogenase (DHODH), and GTP cyclohydrolase 1 (GCH1), which generate radical trapping antioxidant molecules (such as ubiquinol and tetrahydrobiopterin) for ferroptosis suppression (Bersuker et al., 2019; Doll et al., 2019; Kraft et al., 2020; Mao et al., 2021; Soula et al., 2020). Inactivation of these proteins weakens ferroptosis defense systems and promotes ferroptosis.

The dysregulation of ferroptosis has been causally linked to various diseases: excessive ferroptosis contributes to neurodegenerative diseases and ischemia-reperfusion-induced organ injury, whereas insufficient ferroptosis promotes cancer development and metastasis (Jiang et al., 2021; Lei et al., 2022; Stockwell, 2022). In recent years, there has been an exponential growth in the number of published studies on ferroptosis and increasing interest in therapeutically targeting ferroptosis to treat human diseases by ferroptosis inhibitors or inducers (Jiang et al., 2021; Lei et al., 2022; Stockwell, 2022). However, a systematic proteomic analysis of ferroptosis pathways is still lacking, limiting our ability to achieve a comprehensive understanding of underlying biology of ferroptosis and to design therapeutic strategies for treating ferroptosis-associated diseases. In this study, we conducted proteomic analyses of a handful of key proteins involved in ferroptosis regulation, from which we identified choline/ethanolamine phosphotransferase 1 (CEPT1) as a novel binding protein of LPCAT3. CEPT1 is a multi-transmembrane protein localized on the endoplasmic reticulum membrane and a phosphotransferase involved in PL biosynthesis that has a dual enzyme specificity and can use both CDP-choline and CDP-ethanolamine to generate phosphatidylcholine (PC) or phosphatidylethanolamine (PE). We further showed that, contrary to its established role in promoting PL synthesis, CEPT1 plays a role in suppressing ferroptosis. This is potentially achieved by interacting with phospholipases and facilitating the breakdown of specific pro-ferroptotic PUFA-PLs.

## Results

### Proteomic analysis of the protein–protein interaction network involved in ferroptosis pathways

**Figure 1.**
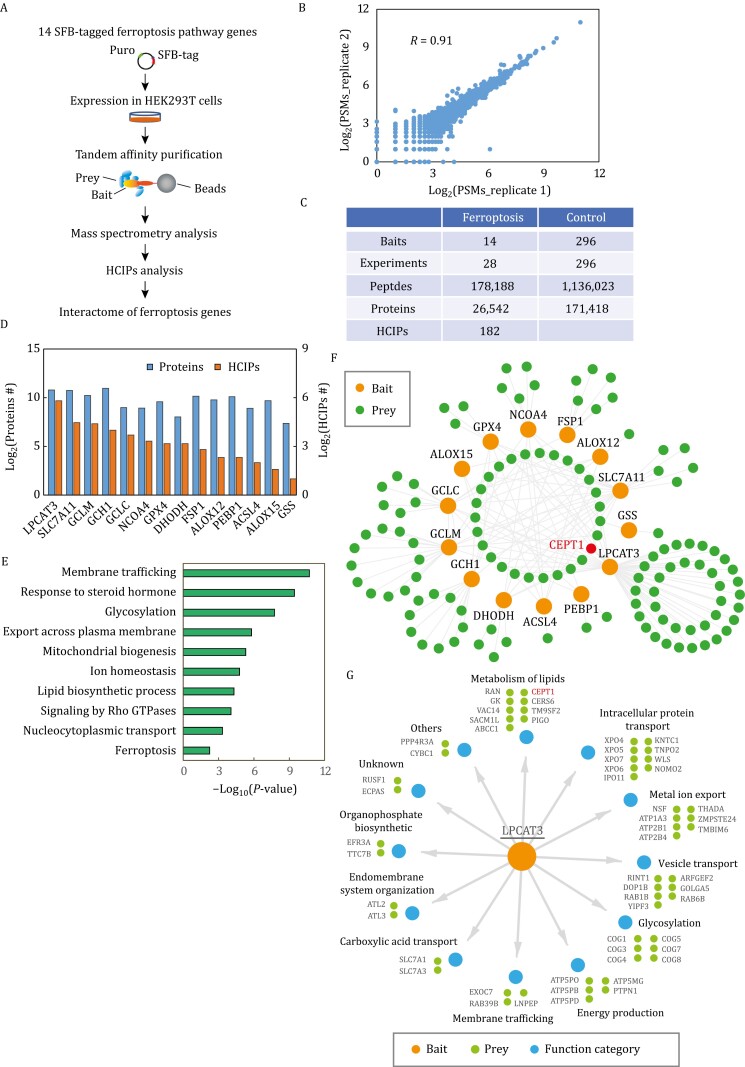
Proteomic analyses of the protein–protein interaction network for ferroptosis pathways. (A) Workflow of mass spectrometry (MS) analysis for tandem affinity purification (TAP) of SFB-tagged protein complexes. Refer to Methods for details. (B) Correlation of the two biological replicates for TAP-MS with each bait. (C) Summary of the TAP-MS datasets for the ferroptosis bait proteins and control bait proteins. (D) HCIPs identified in the purification of each bait protein. (E) Gene Ontology (GO) analysis for the identified HCIPs. (F) Protein–protein interaction network of the 14 proteins involved in ferroptosis pathways. (G) Summary of the Gene Ontology (GO) functional characterization analyzed by Metascape.

To achieve a comprehensive understanding of the protein–protein interaction network involved in ferroptosis regulation, we generated HEK-293T cells stably expressing various human genes fused with SFB triple tags (S protein, FLAG, and streptavidin-binding peptide tags) through viral infection and puromycin selection (Fig. 1A). Fourteen genes encoding proteins in ferroptosis pathways were selected for this study, including genes involved in (1) PUFA-PL synthesis and peroxidation (*ACSL4*, *LPCAT3*, *ALOX12*, *ALOX15*, *PEBP1*), (2) iron metabolism (*NCOA4*), and (3) ferroptosis defense (*SLC7A11*, *GCLC*, *GCLM*, *GSS*, *GPX4*, *GCH1*, *FSP1*, *DHODH*; note that cytosolic GPX4 isoform was used as the bait in our proteomic studies) (Fig. S1A). In this study, we elected to focus on core components of ferroptosis pathways and did not include all proteins that have been identified to regulate ferroptosis. In addition, for technical reasons, we failed to generate SFB vectors or establish stable cell lines for some core ferroptosis genes (such as *POR*). Finally, the ferroptosis field has been rapidly expanding in recent years; new proteins involved in ferroptosis regulation have been constantly identified. Therefore, we acknowledge that the list of proteins studied in this project reflects only a part of ferroptosis pathways.

We validated protein expression in these cell lines and subjected them to tandem affinity purification and mass spectrometry analyses to identify proteins associated with each bait protein (Fig. 1A; see Table S1 for the complete list of identified peptides and proteins). We performed two replicates for each bait protein and observed high correlations between these replicates (Fig. 1B). We then applied SAINTexpress (Teo et al., 2014) to analyze the protein list for high-confidence interacting proteins (HCIPs; proteins with a Bayesian false discovery rate ≤ 0.05) (Fig. S2A). In total, we identified 182 HCIPs for the 14 key ferroptosis regulatory proteins (Fig. 1C, 1D and Table S2). Notably, our proteomic analyses validated several known protein–protein interactions. For example, System X_c_^−^ consists of the transporter subunit SLC7A11 and the regulatory subunit SLC3A2 (Koppula et al., 2021b). Accordingly, SLC3A2 was identified as the top binding protein of SLC7A11 in our analyses (Table S2). Glutamate-cysteine ligase (GCL, the rate-limiting enzyme in glutathione synthesis) is composed of GCLC and GCLM subunits; likewise, GCLC and GLCM were identified as each other’s top binding protein in our study (Table S2). NCOA4 acts as a cargo receptor for ferritinophagy through interaction with ferritin (Mancias et al., 2014); consistently, our proteomic analyses identified both the ferritin heavy chain (FTH1) and ferritin light chain (FTL) as top binding proteins of NCOA4 (Table S2). However, most of the binding proteins identified in our proteomic analyses are novel and have not been described in the literature.

To characterize the biological processes associated with ferroptosis pathways, we conducted Gene Ontology (GO) analysis for these 182 HCIPs (Fig. 1E), which identified enrichment of cellular processes known to be relevant to ferroptosis, such as ferroptosis, the lipid biosynthetic process, and mitochondrial biogenesis [both lipid synthesis and mitochondrial metabolism are important in driving ferroptosis (Jiang et al., 2021; Lei et al., 2022)]; notably, our GO analysis also revealed a significant enrichment of membrane trafficking and nucleocytoplasmic transport processes. We subsequently built a protein-protein interaction network (Fig. 1F), which identified 27 (out of 182) HCIPs that interacted with two or more bait proteins (shown as the dots in the center of the interaction network in Fig. 1F). Further analysis revealed their involvement in diverse cellular processes, such as vesicle transport, intracellular protein transport, energy metabolism, and lipid metabolism (Fig. S2B). These collective findings provide strong evidence suggesting a potential role of intracellular membrane traffic and protein transport in ferroptosis regulation (see Discussion).

Among the bait proteins we analyzed (Fig. S2C), LPCAT3 formed the largest network with the highest number of HCIPs (56 proteins; Fig. 1D), indicating that LPCAT3 is subject to tight regulation or is involved in multiple signaling pathways. Further analyses revealed that LPCAT3-interacting proteins include not only proteins involved in lipid metabolism, as expected but also those regulating intracellular protein transport, vesicle transport, and membrane trafficking, again highlighting a potential functional link to membrane traffic and protein transport (Fig. 1G). Considering that LPCAT3 has been less studied in previous ferroptosis research, we focused on LPCAT3 in our following studies.

### CEPT1 interacts with LPCAT3 and prevents LPCAT3 from undergoing lysosomal degradation

In PL synthesis (Fig. S3A), a fatty acyl group is first activated by acyl-CoA synthetases to form fatty acyl-CoA. Acyl-transferases then successively add two acyl-CoAs to glycerol-3-phosphate to form phosphatidic acid, which is hydrolyzed by lipin to form diacylglycerol. A head group (such as CDP-choline or CDP-ethanolamine) is subsequently added to diacylglycerol by phosphotransferases to form corresponding PLs (such as PC or PE). Acyl-CoA synthetases, acyl-transferases, and phosphotransferases contain family members with specificity for different substrates. For example, ACSL4 and LPCAT3 are the respective acyl-CoA synthetase and acyl-transferase that preferentially utilize PUFAs as their substrates (Liang et al., 2022), explaining their central roles in lipid peroxidation and ferroptosis. However, the exact head groups in PLs that are essential for lipid peroxidation and ferroptosis remain less clear, although previous studies indicated that PEs that contain PUFAs such as arachidonic acids and adrenic acid are main substrates for lipid peroxidation (Kagan et al., 2017). This model would predict that the phosphotransferases involved in the last step of PE synthesis (i.e., add CDP-ethanolamine into diacylglycerol) have a role in driving lipid peroxidation and ferroptosis.

**Figure 2.**
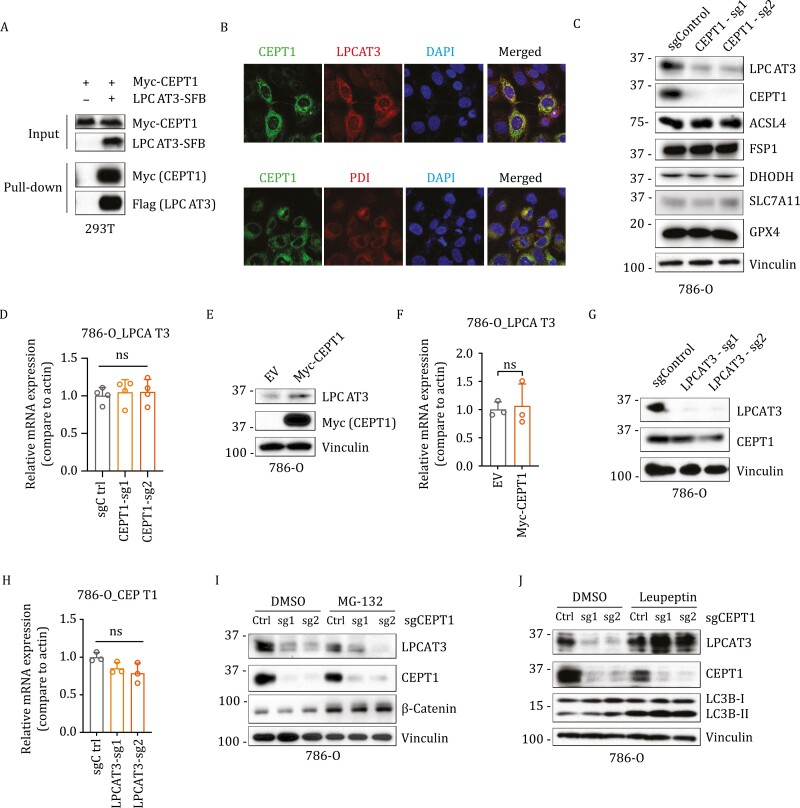
LPCAT3-CEPT1 interaction prevents LPCAT3 from being degraded via lysosomal proteolysis. (A) Co-immunoprecipitation assay was performed with S protein beads (SFB-tagged proteins were used as the baits), and the indicated proteins were detected by Western blot. (B) Immunofluorescence staining was performed to detect the co-localization of CEPT1 with LPCAT3 or the endoplasmic reticulum marker disulfide-isomerase (PDI). (C) Western blot analysis of indicated protein levels in the control (sgControl) and *CEPT1*-knockout (sg1/2) 786-O cells. (D) RT-PCR analysis of the *LPCAT3* mRNA levels in the control (sgControl) and *CEPT1*-knockout (sg1/2) 786-O cells. (E) Western blot analysis of indicated protein levels in 786-O cells overexpressing CEPT1 or transfected with empty vector (EV). (F) RT-PCR analysis of *LPCAT3* mRNA levels in 786-O cells overexpressing CEPT1 or transfected with empty vector (EV). (G) Western blot analysis of indicated protein levels in the control (sgControl) and *LPCAT3*-knockout (sg1/2) 786-O cells. (H) RT-PCR analysis of *CEPT1* mRNA levels in control (sgCtrl) and *LPCAT3*-knockout (sg1/2) 786-O cells. (I) Western blot analysis of indicated protein levels in the control (Ctrl) and *CEPT1*-knockout (sg1/2) 786-O cells treated with DMSO or 5 µmol/L MG-132 for 24 h. (J) Western blot analysis of indicated protein levels in control (Ctrl) and *CEPT1* knockout (sg1/2) 786-O cells treated with DMSO or 40 µmol/L leupeptin for 24 h. ns, not significant.

For these reasons, choline/ethanolamine phosphotransferase 1 (CEPT1) captured our interest as a HCIP of LPCAT3 (Fig. 1G and Table S2). CEPT1 exhibits dual enzyme specificity, capable of utilizing both CDP-choline and CDP-ethanolamine to generate PC and PE (Fig. S3B). We first confirmed the interaction between LPCAT3 and CEPT1 by co-immunoprecipitation (Fig. 2A). PL synthesis occurs mainly on the endoplasmic reticulum membrane. Consistent with this, immunofluorescence analyses revealed co-localization of CEPT1 with LPCAT3 and disulfide-isomerase (PDI, an endoplasmic reticulum marker protein) (Fig. 2B).

We subsequently generated *CEPT1* knockout cell lines in 786-O and HT-1080 cells using CRISPR-Cas9 technology. Of note, we chose 786-O and HT-1080 cells in our functional studies because these two cell lines are widely used in ferroptosis research; in addition, both cell lines exhibit substantial expression levels of CEPT1 and LPCAT3 (Fig. S4A), facilitating the execution of various functional studies within these cell lines. Notably, *CEPT1* deletion markedly reduced protein levels of LPCAT3 without affecting its mRNA levels or expression levels of other key ferroptosis regulators, such as ACSL4, SLC7A11, GPX4, FSP1, and DHODH (Figs. 2C, 2D and S4B–M). It should be noted that our proteomic analyses identified SLC7A11 as the other bait protein that interacted with CEPT1. This interaction was further validated by co-immunoprecipitation (Fig. S4N); however, CEPT1 deletion did not appear to affect SLC7A11 levels or its function in cystine uptake (Figs. 2C, S4B and S4O). Conversely, ectopic expression of CEPT1 escalated LPCAT3 protein levels without affecting its mRNA levels (Fig. 2E, 2F, S4P and S4Q). By contrast, *LPCAT3* deletion did not affect protein or mRNA levels of CEPT1 (Fig. 2G and 2H). We then tested whether *CEPT1* deletion promotes LPCAT3 protein degradation. There are two main protein degradation pathways, namely the ubiquitin-proteasome pathway and the lysosomal degradation pathway. We found that treatment with the lysosomal inhibitor leupeptin or bafilomycin A1, but not the proteasome inhibitor MG-132, restored LPCAT3 protein levels in *CEPT1* knockout cells (Figs. 2I, 2J, S4R and S4S; MG-132 treatment increased protein levels of β-catenin and p53 as positive controls). We further showed that supplementation of arachidonic acid (a type of PUFA) rendered cells more sensitive to ferroptosis but failed to restore LPCAT3 protein levels in *CEPT1* knockout cells (Fig. S4T and S4U). Taken together, our data reveal that CEPT1 interacts with and stabilizes LPCAT3 by preventing it from undergoing lysosomal degradation.

### CEPT1 regulates ferroptosis

**Figure 3.**
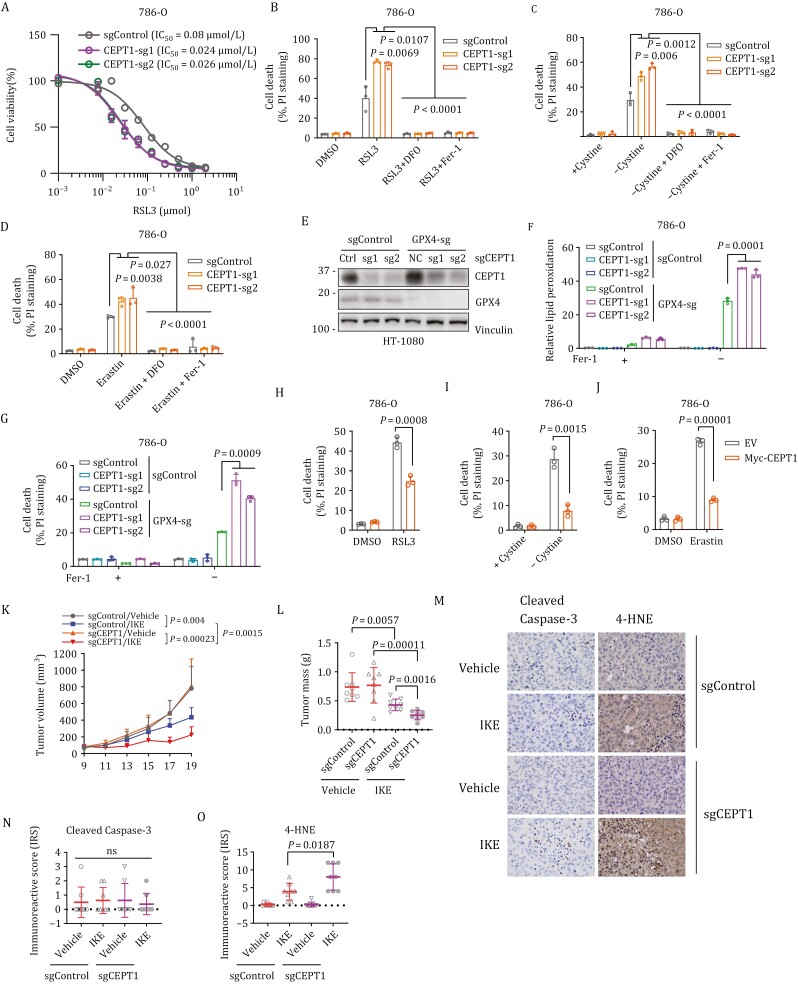
CEPT1 inhibits ferroptosis. (A) Cell viability was measured in the control (sgControl) and *CEPT1*-knockout (CEPT1-sg1 and CEPT1-sg2) 786-O cells treated with different doses of RSL3 for 24 h. (B) Cell death was measured by propidium iodide (PI) staining in the control (sgControl) and *CEPT1*-knockout (sg1/2) 786-O cells treated with DMSO, 50 nmol/L RSL3, 100 µmol/L deferoxamine (DFO), or 10 µmol/L ferrostatin-1 (Fer-1) for 24 h. (C) Cell death was measured by PI staining in the control (sgControl) and *CEPT1*-knockout (sg1/2) 786-O cells cultured in cystine-containing (+Cystine) or cystine-free (−Cystine) medium with or without 100 µmol/L DFO or 10 µmol/L Fer-1 for 24 h. (D) Cell death was measured by PI staining in the control (sgControl) and *CEPT1*-knockout (sg1/2) 786-O cells treated with DMSO, 10 µmol/L erastin, 100 µmol/L DFO, or 10 µmol/L Fer-1 for 24 h. (E) Western blot analysis of indicated protein levels in control (sgCtrl) and *CEPT1*-*GPX4* single and double knockout HT-1080 cells. (F and G) Relative lipid peroxidation and cell death by PI staining in the control (sgCtrl) and *CEPT1*-*GPX4* single or double knockout HT-1080 cells with or without 5 µmol/L ferrostatin-1 (Fer-1) for 48 (F) or 72 (G) h. (H) Cell death was measured by PI staining in 786-O cells overexpressing CEPT1 or transfected with empty vector (EV) treated with DMSO or 50 nmol/L RSL3 for 24 h. (I) Cell death was measured by PI staining in 786-O cells overexpressing CEPT1 or transfected with empty vector (EV) cultured in cystine-containing (+Cystine) or cystine-free (−Cystine) medium for 24 h. (J) Cell death was measured by PI staining in 786-O cells overexpressing CEPT1 or transfected with empty vector (EV) with treatment of DMSO or 10 µmol/L erastin for 24 h. (K) Tumor volumes over time in control (sgControl) and CEPT1-knockout (sgCEPT1) HT-1080 cells-derived xenografts under the indicated treatments. (L) End-point weights of HT-1080 xenograft tumors with indicated genotypes treated with IKE or vehicle. (M–O) Representative immunochemical images (M) from HT-1080 xenograft tumors with indicated genotypes treated with IKE or vehicle and corresponding immunoreactive scores of cleaved caspase-3 (N) or 4-HNE (O).

CEPT1’s roles in synthesizing PE and stabilizing LPCAT3 suggest that, similar to *LPCAT3* knockout cells (Fig. S5A), *CEPT1* deficient cells would exhibit ferroptosis resistance phenotypes. Surprisingly, we found that *CEPT1* deletion sensitized different cell lines to RSL3-, cystine starvation- or erastin-induced lipid peroxidation and ferroptosis, and this sensitization was largely abolished by the ferroptosis inhibitor ferrostatin-1 (Fer-1) or the iron chelator deferoxamine (DFO) (Figs. 3A–D and S5B–G). We also generated *CEPT1* and *GPX4* single and double knockout HT-1080 cells (Fig. 3E). These cells were generated and passaged in the medium supplemented with Fer-1 to maintain their survival. Subsequent removal of Fer-1 significantly increased lipid peroxidation and cell death in *GPX4* knockout cells, but not in control or *CEPT1* knockout counterparts; importantly, *CEPT1* deletion further enhanced lipid peroxidation and cell death in *GPX4* knockout cells (Fig. 3F and 3G). These data reinforced our observations derived from RSL3 treatment conditions. Conversely, CEPT1 overexpression suppressed ferroptosis triggered by these ferroptosis inducers (Figs. 3H–J and S5H–J).

We further investigated whether *CEPT1* deletion enhances the sensitivity of xenograft tumors to imidazole ketone erastin (IKE), an erastin analogue and a suitable inducer of ferroptosis for *in vivo* treatment (Zhang et al., 2019b). We observed that *CEPT1* deletion did not impact the growth of HT-1080 xenograft tumors but rendered the tumors more susceptible to IKE treatment; consequently, *CEPT1* knockout tumors treated with IKE exhibited reduced tumor growth compared to control tumors subjected to IKE treatment (Fig. 3K and 3L). Immunohistochemical analyses revealed that neither *CEPT1* deletion nor IKE treatment appeared to affect the staining of the apoptosis surrogate, cleaved Caspase-3 (Fig. 3M and 3N). As expected, IKE treatment increased the staining of 4-HNE, a marker of lipid peroxidation; moreover, the staining of 4-HNE was further intensified in *CEPT1* knockout tumor samples treated with IKE, compared to control tumors treated with IKE (Fig. 3M and 3O). Importantly, the administration of IKE did not impact the weight of the mice in these animal studies (Fig. S5K). These *in vivo* data provided additional support for a ferroptosis-suppressive function of CEPT1.

As noted above, CEPT1 catalyzes the generation of both PE and PC from diacylglycerol in the glycerophospholipid biosynthesis pathway; PE and PC can also be synthesized from diacylglycerol by ethanolamine phosphotransferase 1 (EPT1) and choline phosphotransferase 1 (CHPT1 or CPT1), respectively (Fig. S6A). We therefore studied whether depleting EPT1 or CHPT1 exerts effects on ferroptosis similar to those of CEPT1 loss. Of note, EPT1 was also identified as a binding protein of LPCAT3 from our proteomic studies (Table S1), although it was not ranked as a HCIP in subsequent analyses. We confirmed the interaction between LPCAT3 and EPT1 by co-immunoprecipitation (Fig. S6B). Notably, knocking down *EPT1* by shRNA or knocking out *CHPT1* by CRISPR-Cas9 rendered cells markedly resistant to ferroptosis triggered by different ferroptosis inducers (Fig. S6C–H). These data are consistent with the roles of EPT1 and CHPT1 in generating PC and PE, which act as substrates for lipid peroxidation; therefore, deficiency of *EPT1* or *CHPT1* leads to ferroptosis resistance. In addition, knocking down *EPT1* or knocking out *CHPT1* did not apparent affect LPCAT3 protein levels (Fig. S6D and S6G). This further highlights the unexpected role of CEPT1 in regulating ferroptosis.

Collectively, our data suggest that CEPT1 functions as a ferroptosis suppressor, as its deletion renders cells more susceptible to ferroptosis. Nevertheless, it appears that its role in ferroptosis suppression cannot be attributed to its function in generating PLs or its impact on stabilizing LPCAT3 (as *LPCAT3* deletion and *CEPT1* deletion showed opposite phenotypes in regulating ferroptosis).

### CEPT1 inhibition of ferroptosis is independent of its enzymatic activity but dependent on ACSL4

**Figure 4.**
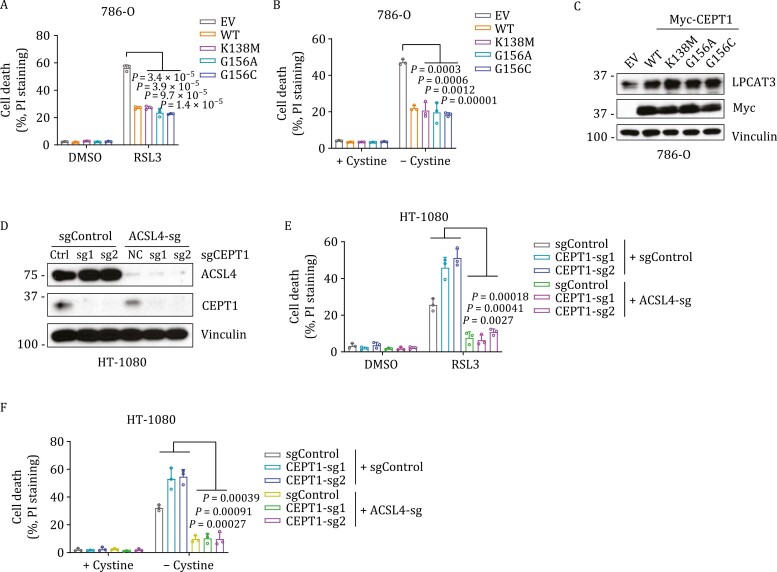
CEPT1 inhibition of ferroptosis is independent of its enzymatic activity but dependent on ACSL4. (A) Cell death was measured by propidium iodide (PI) staining in 786-O cells overexpressing CEPT1 wild-type (WT) or indicated mutants or transfected with empty vector (EV) with treatment of DMSO or 50 nmol/L RSL3 for 24 h. (B) Cell death was measured by PI staining in the 786-O cells overexpressing CEPT1 WT or indicated mutants or transfected with EV cultured in cystine-containing (+Cystine) or cystine-free (−Cystine) medium for 24 h. (C) Western blot analysis of indicated protein levels in the 786-O cells overexpressing CEPT1 WT or indicated mutants or transfected with EV. (D) Western blot analysis of indicated protein levels in control (sgCtrl) and *CEPT1*-*ACSL4* single or double knockout HT-1080 cells. (E) Cell death was measured by PI staining in the control (sgCtrl) and *CEPT1*-*ACSL4* single or double knockout HT-1080 cells treated with DMSO or 50 nmol/L RSL3 for 24 h. (F) Cell death was measured by PI staining in control (sgCtrl) and *CEPT1*-*ACSL4* single or double knockout HT-1080 cells cultured in cystine-containing (+Cystine) or cystine-free (−Cystine) medium for 24 h.

Considering CEPT1 is an enzyme, we next studied whether its ferroptosis-inhibitory effect is dependent on its enzymatic activity. To this end, we generated several enzyme-dead mutants of CEPT1 identified from a previous study (Henneberry et al., 2002). Interestingly, these mutants exhibited suppression of ferroptosis similar to that of wild-type CEPT1 (Fig. 4A and 4B). Furthermore, ectopic expression of these mutants and wild-type CEPT1 at similar levels increased LPCAT3 protein levels to the same extent (Fig. 4C). These data suggest that CEPT1 inhibits ferroptosis (and maintains LPCAT3 protein stability) independent of its enzymatic activity.

ACSL4 has an essential role in ferroptosis execution whereby it promotes the synthesis of PUFA-PLs (Dixon et al., 2015; Doll et al., 2017; Yuan et al., 2016). To study whether *CEPT1* deficiency-induced ferroptosis is dependent on ACSL4, we deleted *ACSL4* in control and *CEPT1*-knockout cells by CRISPR-Cas9 (Fig. 4D). Knocking out *ACSL4* did not affect CEPT1 protein levels, and conversely, knocking out *CEPT1* did not affect ACLS4 protein levels (Fig. 4D). We showed that deleting *ACSL4* blocked ferroptosis induction in both control and *CEPT1*-knockout cells and thereby abolished the ferroptosis sensitization phenotype in *CEPT1*-knockout cells (Figs. 4E, 4F and S7A). Collectively, our data indicate that CEPT1 inhibits ferroptosis independent of its enzymatic activity but in an ACSL4-dependent manner.

### CEPT1 inhibits ferroptosis partly through phospholipases

To study how CEPT1 inhibits ferroptosis, we performed tandem affinity purification followed by mass spectrometry to identify CEPT1 binding proteins (Table S3). We identified multiple phospholipases, including phospholipase A1 (PLA1) proteins (DDHD1, DDHD2, ABHD3), and phospholipase A2-activating protein (PLAA) as CEPT1-associated proteins (Table S3; of note, LPCAT3 was also identified as a CEPT1-interacting protein in this analysis). PLA1 and PLA2 proteins cleave the sn-1 and sn-2 positions of the glycerol moieties of PLs to generate free fatty acids and 2-acyl lysophospholipids or 1-acyl lysophospholipids, respectively (Fig. S7B). Notably, iPLA2β (a PLA2 member)-mediated breaking-down of PLs has been shown to suppress ferroptosis (Beharier et al., 2020; Chen et al., 2021a; Sun et al., 2021).

**Figure 5.**
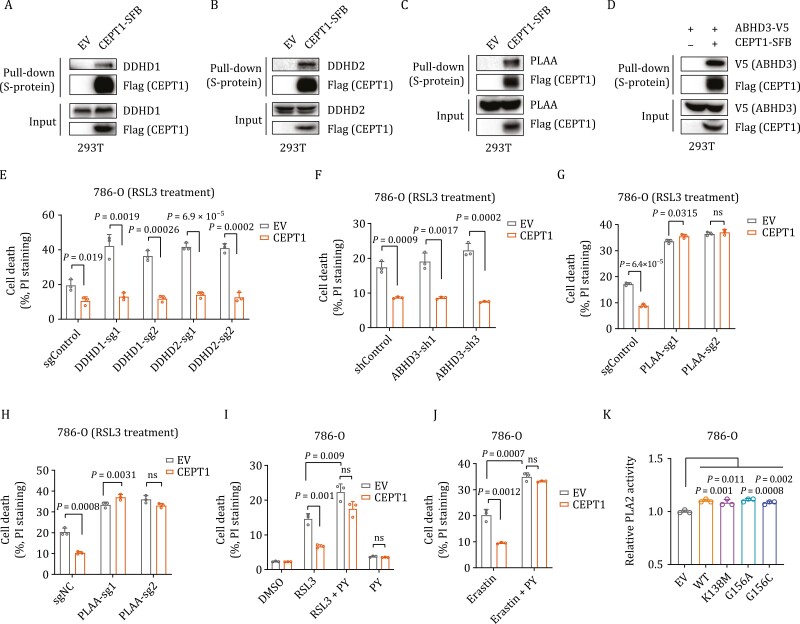
Ferroptosis inhibition by CEPT1 is partially dependent on phospholipases. (A–D) Pulldown assays were performed with S protein beads (SFB-tagged proteins were used as the baits), and the indicated proteins were detected by WB. (E) Cell death was measured by propidium iodide (PI) staining in control (sgControl), *DDHD1*-knockout, or *DDHD2*-knockout (sg1/2) 786-O cells overexpressing CEPT1 or transfected with empty vector (EV) treated with 50 nmol/L RSL3 for 24 h. (F) Cell death was measured by PI staining in control (shControl) and *ABHD3*-knockdown (sh1/3) 786-O cells overexpressing CEPT1 or transfected with EV treated with 50 nmol/L RSL3 for 24 h. (G and H) Cell death was measured by PI staining in control (sgControl) and *PLAA*-knockout (sg1/2) 786-O cells overexpressing CEPT1 or transfected with EV treated with 50 nmol/L RSL3 (G) or 10 µmol/L erastin (H) for 24 h. (I and J) Cell death was measured by PI staining in 786-O cells overexpressing CEPT1 or transfected with EV treated with DMSO, 50 nmol/L RSL3 (I), or 10 µmol/L erastin (J) with 1 µmol/L pyrrophenone (PY) or indicated combinations for 24 h. (K) PLA2 enzyme activities were measured in 786-O cells overexpressing CEPT1 WT or indicated mutants or transfected with an empty vector (EV).

We confirmed the interaction between CEPT1 and these proteins (Fig. 5A–D). We then depleted these proteins in control and CEPT1-overexpressing cells by shRNAs or CRISPR-Cas9 (Fig. S7C–F). We showed that depleting DDHD1 or DDHD2 (but not ABHD3) sensitized cells to RSL3-induced ferroptosis; however, CEPT1 overexpression suppressed ferroptosis in both control and DDHD1-, DDHD2-, or ABHD3-depleted cells (Fig. 5E and 5F), suggesting that CEPT1 inhibits ferroptosis independent of these proteins. PLAA depletion sensitized cells to RSL3- or erastin-induced ferroptosis; importantly, the ferroptosis-suppressive effect of CEPT1 overexpression was abolished in *PLAA*-deficient cells (Fig. 5G and 5H). Likewise, treatment with pyrrophenone [PY, a pharmacological inhibitor of cytosolic PLA2 (cPLA2, which can be activated by PLAA)] promoted RSL3- or erastin-induced ferroptosis, and the effect of CEPT1 overexpression on suppressing ferroptosis was largely compromised under PY treatment conditions (Fig. 5I and 5J). Finally, we showed that both CEPT1 wild-type and mutants interacted with PLAA (Fig. S7G) and enhanced PLA2 activity (Fig. 5K), which correlated with ferroptosis-suppressive effects of these mutants (Fig. 4A and 4B). Taken together, these results reveal that CEPT1 interacts with PLAA and that the ferroptosis-inhibitory effect of CEPT1 is at least partly mediated through PLAA, which is consistent with the ferroptosis-suppressing effect of phospholipases.

### CEPT1 downregulates PUFA-containing PLs and triacylglycerols

**Figure 6.**
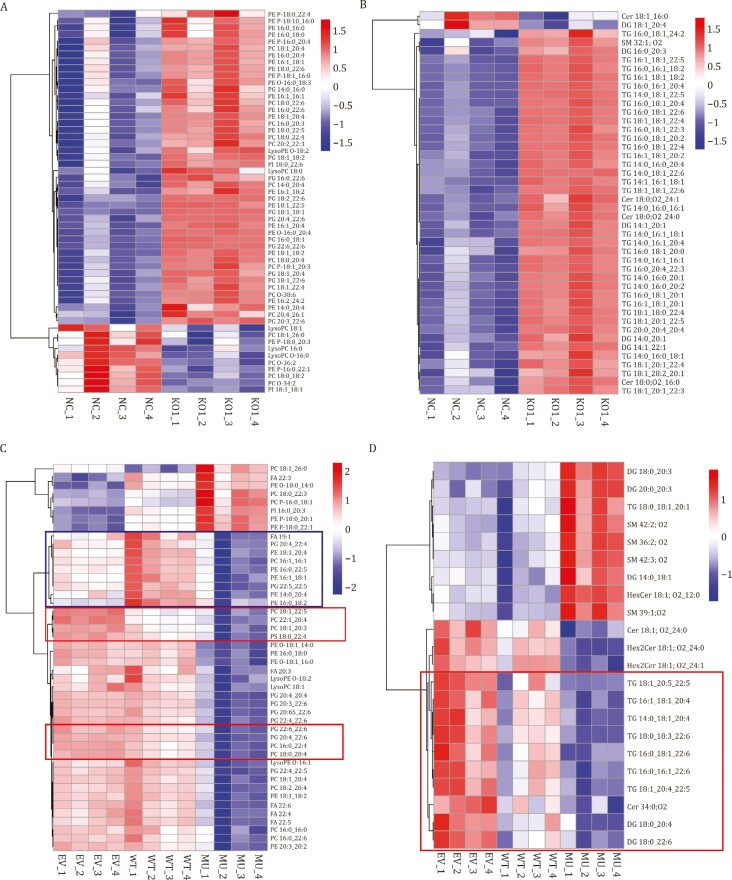
CEPT1 downregulates PUFA-containing phospholipids and triacylglycerols. (A and B) Heat maps of significantly changed phospholipid (A) or sphingolipids and glycolipids (B) (One-way ANOVA; FDR-corrected *P*-value < 0.01; *n* = 4 repeats) in the control (NC) and CEPT1-knockout (KO) HT-1080 cells. (C and D) Heat maps of significantly changed phospholipid (C) or sphingolipids and glycolipids (D) (One-way ANOVA; FDR-corrected *P*-value < 0.01; *n* = 4 repeats) in 786-O cells overexpressing CEPT1 WT or K138M mutant (MU) or transfected with an empty vector (EV). Each row represents *z*-score-normalized intensities of the detected lipid species. Each column represents a sample. PE, phosphatidylethanolamine; PE P, plasmalogen PE; PE O, ether-linked PE; LysoPE, lysophosphatidylethanolamine; PC, phosphatidylcholine; PC P, plasmalogen PC; PC O, ether-linked PC; LysoPC, lysophosphatidylcholine; PG, phosphatidylglycerol; PI, phosphatidylinositol; PS, phosphatidylserine; Cer, ceramide; DG, diacylglycerol; TG (also named TAG), triacylglycerol; SM, sphingomyelin.

The aforementioned data prompted us to characterize lipid profile alterations caused by CEPT1 depletion or overexpression. Untargeted lipidomic analysis in control (NC) and *CEPT1* knockout (KO) HT-1080 cells (Figs. 6A, 6B and S8A) revealed that *CEPT1* deletion reduced the levels of certain PE and PC species, which is consistent with its enzyme activity involved in PC and PE generation. Surprisingly, *CEPT1* deletion resulted in even more pronounced increases in the lipidome, including many PUFA-containing PLs and PUFA-containing triacylglycerols (TAGs). Conversely, we also conducted untargeted lipidomic analyses in 786-O cells expressing empty vector (EV), CEPT1 wild-type (WT), and CEPT1 K138M enzyme-dead mutant (MU) (Figs. 6C, 6D and S8B). We identified several PC and PE species whose levels were elevated by CEPT1 WT overexpression but reduced by CEPT1 MU overexpression (see the top box in Fig. 6C). This observation is possibly mediated by the canonical function of CEPT1 in PC and PE generation (the decreased PC and PE levels resulting from CEPT1 MU overexpression possibly reflect the dominant-negative effect of the CEPT1 enzyme-dead mutant). Irrespective of the exact mechanisms driving alterations in these lipid species, they do not offer an explanation for the observed similar ferroptosis resistance phenotypes in both CEPT1 WT and MU cells. Additionally, we identified multiple PUFA-containing PLs and TAGs that were decreased upon overexpression of both CEPT1 WT and mutant (see the two lower boxs in Fig. 6C and the box in Fig. 6D); these lipid species associate with and therefore likely mediate the ferroptosis resistance phenotypes in cells with overexpression of CEPT1 WT or MU.

Together, our lipidomic analyses demonstrated that *CEPT1* deletion increases whereas overexpression of CEPT1 wild-type or mutant reduces the levels of PUFA-PLs, which correlates with CEPT1’s ability to suppress ferroptosis. This function likely reflects a non-canonical role of CEPT1 that is independent of its function in PC and PE generation. The impact of CEPT1 on decreasing PUFA- PLs potentially relates to its interaction with phospholipases and promotion of phospholipase activity (to break down PUFA-PLs).

## Discussion

In this study, we analyzed the interaction network of 14 key regulators of ferroptosis pathways. While the rapid expansion of ferroptosis research and other technical reasons prevented us from studying the full repertoire of proteins involved in ferroptosis pathways, the identification of 182 HCIPs in the current study still greatly expanded our understanding of ferroptosis regulation and will provide important insights for future functional studies. For example, while different subcellular compartments (including the plasma membrane, mitochondria, endoplasmic reticulum, and peroxisomes) have been shown to participate in ferroptosis execution (Stockwell, 2022), how these compartments/cellular organelles coordinate and communicate to ensure an appropriate “live or die” decision for the cell remains poorly understood. Our proteomic analyses revealed a significant enrichment of proteins involved in membrane trafficking and intracellular transport, which will inspire future investigations to study the role of protein transport and organelle communication in regulating ferroptosis. For example, considering that both LPCAT3 and CEPT1 are localized on the endoplasmic reticulum, it is plausible that certain proteins interacting with LPCAT3 and/or CEPT1 are involved in modulating their trafficking between various subcellular compartments. This dynamic interplay may potentially contribute to the regulation of ferroptosis.

**Figure 7.**
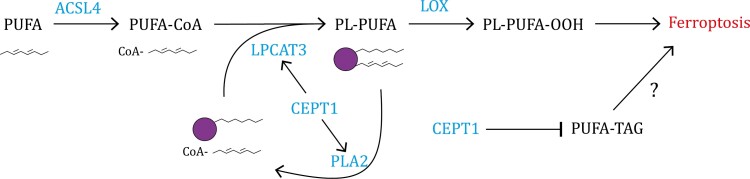
Working model depicting how CEPT1 regulates ferroptosis. See Discussion for a detailed description.

In the current study, we focused on CEPT1, which was identified as a HCIP of LPCAT3 in our proteomic analyses. Both LPCAT3 and CEPT1 are known to participate in PL synthesis: while LPCAT3 catalyzes the insertion of PUFA-CoA into PLs, CEPT1 mediates the addition of the head groups (CDP-choline and CDP-ethanolamine) into PLs to generate PCs and PEs. We further confirmed that LPCAT3 and CEPT1 co-localize at the endoplasmic reticulum, an organelle that is essential for PL synthesis. Interestingly, our data showed that CEPT1 is also required for maintaining LPCAT3 protein stability by preventing LPCAT3 from lysosomal degradation, which is independent of CEPT1’s enzymatic activity. By stabilizing LPCAT3, CEPT1 is expected to exert a pro-ferroptosis role. In addition, other enzymes catalyzing the addition of head groups into PLs, such as EPT1 and CHPT1, were found to promote ferroptosis as expected. Therefore, it was surprising that our study revealed an anti-ferroptosis function of CEPT1 by demonstrating that *CEPT1* deficiency sensitized cells to ferroptosis. Together, these data suggest that CEPT1 has an anti-ferroptosis function; nevertheless, this effect may be partially counterbalanced by its involvement in PL generation and the promotion of LPCAT3 protein stability (Fig. 7), which likely contributes to the moderate ferroptosis sensitization phenotypes in *CEPT1* deficient cells.

Several lines of evidence suggest that CEPT1 inhibits ferroptosis at least partly through interacting with PLAA (an activator of cPLA2) and promoting phospholipases’ function to cleave PUFA-PLs (Fig. 7). First, we showed that both CEPT1 wild-type and enzyme-dead mutants interacted with PLAA, increased phospholipase activity, and suppressed ferroptosis. In addition, the overexpression of CEPT1 wild-type and mutant decreased the levels of multiple PUFA-PLs, consistent with PLA’s function in breaking down PUFA-PLs. Finally, the suppression of ferroptosis by CEPT1 overexpression was largely compromised in *PLAA*-knockout cells and under cPLA2 inhibition conditions. Further studies are required to understand the underlying mechanisms by which CEPT1 regulates phospholipases and suppresses ferroptosis in an enzymatic-independent manner.

Our study also raises the question of why CEPT1 has such multifaceted functions in lipid metabolism, which is unusual among lipid metabolism enzymes involved in ferroptosis regulation. Of note, while PUFA-PLs provide substrates for lipid peroxidation and promote ferroptosis, they are also required for normal cellular functions, including maintaining membrane fluidity and cell signaling (Rohrig and Schulze, 2016). We reason that perhaps CEPT1 has evolved different (enzyme-dependent and -independent) functions to fine-tune PUFA-PL levels for cellular homeostasis. On one hand, CEPT1 both acts as a phosphotransferase and governs LPCAT3 protein stability to promote the synthesis of PUFA-containing PEs and PCs, which are important for cellular signaling and membrane structural maintenance. On the other hand, CEPT1 decreases certain PUFA-containing lipid species, such as PUFA-PLs and PUFA-TAGs, to minimize their toxic effects in driving lipid peroxidation and ferroptosis (Fig. 7). Together, we propose that CEPT1 acts as an important hub in coordinating lipid metabolism with cell viability and homeostasis.

This model is consistent with the intricate nature of our lipidomic data. In contrast to its established role in promoting PE and PC synthesis, we did not observe a consistent overall increase or decrease of these lipid species in our lipidomic data from cells with either overexpression or deficiency of CEPT1. This discrepancy likely reflects CEPT1’s dual role in promoting the generation of certain PLs while concurrently facilitating the breakdown of others. In addition, in the absence of CEPT1, compensatory mechanisms may be at play, wherein EPT1 and CHPT1 potentially drive PE and PC synthesis, resulting in a limited decrease in the number of PE and PC species in *CEPT1* knockout cells. Notably, *EPT1* knockdown or *CHPT1* knockout cells exhibit substantial ferroptosis resistance phenotypes. Further lipidomic analyses in these cells could provide valuable insights into the intricate interplay of these enzymes in cellular lipid metabolism and the regulation of ferroptosis.

In summary, our proteomic analysis of the protein–protein interaction network involved in ferroptosis identifies many previously unknown interactions potentially involved in this intriguing cell death mechanism and provides an important resource for future studies to further understand ferroptosis mechanisms. Most notably, we identified CEPT1 as a LPCAT3-interacting protein and an important regulator of lipid metabolism and ferroptosis.

## Materials and methods

### Cell culture studies

All cell lines were obtained from the American Type Culture Collection and free of *Mycoplasma* contamination (tested by the vendor). No cell line used in this study has been found in the International Cell Line Authentication Committee database of commonly misidentified cell lines, based on short tandem repeat profiling performed by the vendor. Normally, cells were cultured in Dulbecco modified Eagle’s medium (DMEM) with 10% [volume/volume (*v*/*v*)] fetal bovine serum and 1% (*v*/*v*) penicillin/streptomycin in a cell incubator containing 5% CO_2_ air atmosphere with 37°C. For cystine deprivation experiments, cells were cultured in cystine-free DMEM supplemented with dialyzed fetal bovine serum as previously described (Liu et al., 2020; Zhang et al., 2018). The cystine-free DMEM was customized by Athena Environmental Sciences.

### Constructs and reagents

Plasmids encoding the indicated genes were obtained from the Human ORFeome v5.1 library or purchased from Open Biosystems. For tandem affinity purification analysis, all entry clones were subsequently recombined into lentiviral Gateway-compatible destination vectors for the expression of C-terminal SFB-tagged fusion proteins. CRISPR-mediated knockout plasmids containing guide RNAs targeting *CEPT1*, *LPCAT3*, *ACSL4*, *CHPT1*, *DDHD1*, *DDHD2*, *GPX4* and *PLAA* were generated in lentiCRISPR v2 (Addgene, #52961) as previously described (Koppula et al., 2021a; Lei et al., 2021). The shRNA constructs targeting *EPT1* were obtained from the Functional Genomics Core Facility of The University of Texas MD Anderson Cancer Center. A series of mutant Myc-CEPT1 constructs were generated by polymerase chain reaction (PCR) mutagenesis using a Site-Directed Mutagenesis Kit (New England Biolabs, #E0554S) for amino acid substitutions according to the manufacturer’s instructions. All constructs were confirmed by DNA sequencing. The sequences of gRNAs and shRNA used in this study are listed in Table S4. Ferroptosis inducer (1S,3R)-RSL3 (#19288), erastin (#17754), arachidonic acid (#90010), and pyrrophenone (#13294) were from Cayman Chemical. The following reagents were obtained from Sigma-Aldrich: deferoxamine mesylate salt (#D9533), ferrostatin-1 (#SML0583), MG-132 (#474787), leupeptin (#L2884), bafilomycin A1 (#SML1661).

### Stable cell line generation and CRISPR/Cas9-mediated gene knockout

Cell lines with stable expression of constructs or target genes were generated as previously described (Liu and Gan, 2016; Liu et al., 2016). Briefly, HEK293T cells were transfected with lentiviral constructs together with the psPAX.2 and pMD2.G third-generation lentiviral packaging system using Lipofectamine 2000 reagent (Life Technologies) according to the manufacturer’s instructions. After 72 h, the media containing lentivirus particles were collected and filtered, and then the target cell lines were infected with polybrene transfection reagent (8 µg/mL). At 24 h post-infection, spent media were replaced with fresh media containing puromycin (2 µg/mL) for culturing for 1–2 weeks, and stable cell lines were obtained with successful transduction. For the generation of *GPX4* knockout cell lines, 5 µmol/L ferrostatin-1 was added to the culture media to maintain the survival of *GPX4* knockout cells.

### Tandem affinity purification of SFB-tagged protein complexes

SFB-tandem affinity purification was done in accordance with our previous work (Chen et al., 2021b). HEK293T cells with SFB-tagged key regulating genes of ferroptosis were harvested and subjected to lysis NETN buffer (100 mmol/L NaCl, 1 mmol/L EDTA, 20 mmol/L Tris-HCl, and 0.5% Nonidet P-40) with protease cocktail (Sigma-Aldrich) for 20 min at 4°C. The supernatant was collected after centrifugation at 16,000 ×*g* for 20 min and then incubated with streptavidin-conjugated beads (Thermo Fisher Scientific) for 2 h at 4°C. A total of 3 mg protein lysates was used for affinity purification in each proteomic experiment. After three washings with NETN buffer, the SFB-tagged samples were eluted with NETN buffer plus 2 mg/mL biotin. The elutes were then incubated to S-protein agarose (VWR International) for 2 h at 4°C. The beads were washed three times with NETN buffer and boiled in sodium dodecyl sulfate-polyacrylamide gel electrophoresis (SDS-PAGE) loading buffer. The sample was subjected to SDS-PAGE and stained by Coomassie brilliant blue.

### Mass spectrometry analysis

The sample in the gel was excised, destained, and digested by trypsin (Promega Corporation) at 37°C overnight. The peptides were extracted from the gel, vacuum-dried, and then reconstituted in the mass spectrometry (MS) loading solution (2% acetonitrile and 0.1% formic acid). The MS analysis was run with nano-reverse-phase high-performance liquid chromatography and the Q Exactive HF MS system (Thermo Fisher Scientific). The sample was eluted with acetonitrile gradient from 5% to 35% for 60 min at a flow rate of 300 nL/min. The MS machine was set to positive ion mode, data-dependent MS with a scanning range of 350–1,200 m/z and resolution at 60,000 at *m*/*z* 400, and one full scan was followed by up to 20 MS/MS scans.

The MS raw data were submitted to Proteome Discoverer 2.2 (Thermo Fisher Scientific) and searched by Mascot 2.5 (Matrix Science) with the database for *Homo sapiens* downloaded from Uniprot (20,352 entries in total, July 2020). The variable modifications included oxidation for methionine and carboxyamidomethyl. The mass tolerance was 10 ppm for the precursor and 0.02 Da for the production. Two missed cleavages tolerance of trypsin was applied. Common contaminant proteins were removed from the output protein list. We applied SAINTexpress (version 3.6.3) to filter the list by comparison to the controls (296 tandem affinity purification-MS experiments using different bait genes from our previous publications (Chen et al., 2016, 2020b, 2021b; Li et al., 2015, 2016a, 2016b; Srivastava et al., 2018; Wang et al., 2020a, 2020b, 2022; Zhang et al., 2019a, 2022) as well as unpublished studies; we selected those genes that have not been reported to have any known function directly related to ferroptosis pathways). SAINTexpress Bayesian false discovery rate ≤0.05 was chosen as the cutoff for the HCIPs list. We used Cytoscape to generate the interactome network and Metascape to analyze the functional characterization (Zhou et al., 2019).

### Pulldown, immunoprecipitation, and Western blot

Pulldown and immunoprecipitation were conducted as previously described (Gan et al., 2005; Lin et al., 2014b). Western blot was conducted as previously described with modifications (Chauhan et al., 2019; Xiao et al., 2017). Cells were harvested and lysed in NP40 buffer followed by centrifugation. The supernatant was combined with NuPAGE LDS Sample Buffer (4X) (Life Technologies, #NP0007) and kept at room temperature for 30 min before SDS-PAGE analysis. The primary antibodies and concentrations used for Western blot were as follows: Myc tag (1:2,000, Cell Signaling Technology, #2276S); Flag tag (1:2,000, Cell Signaling Technology, #14793S); LPCAT3 (1:1000, Abcam, #ab232958); CEPT1 (1:1000, Proteintech, #20496-1-AP); ACSL4 (1:2,000, Santa Cruz Biotechnology, #sc-271800); FSP1/AIFM2 (1:1000, Proteintech, #20886-1-AP); DHODH (1:1000, Proteintech, #14877-1-AP); GPX4 (1:1000, R&D systems, #MAB5457); vinculin (1:5,000, Sigma-Aldrich, #V4505); β-catenin (1:1000, Cell Signaling Technology, #9562s); LC3B (1:2,000, Cell Signaling Technology, #3868s); p53 (1:2,000, Santa Cruz Biotechnology, #sc-126); CHPT1 (1:2,000, Thermo Fisher Scientific, #PA5-23695); DDHD1 (1:2,000, Thermo Fisher Scientific, #A305-756A-M); DDHD2 (1:2,000, Proteintech, #25203-1-AP); PLAA (1:2,000, Proteintech, #12529-1-AP); and V5 (1:2,000, Cell Signaling Technology, #13202S). Uncropped Western blots are provided in Supplementary Materials.

### Cystine uptake

Cystine uptake assay was performed using L-[1,2,1ʹ,2ʹ-^14^C]-cystine (PerkinElmer) as previously described (Lee et al., 2024). Briefly, cells in a 12-well plate were washed once with PBS and fresh media containing 0.1 μCi L-[1,2,1ʹ,2ʹ-^14^C]-cystine were added to the wells. After incubation for 2 h, the cells were washed twice with PBS and lysed in 0.1 mmol/L NaOH solution. Then the radioactivity (dpm) was measured using a Tri-Carb liquid scintillation analyzer (PerkinElmer, model 4810TR) in the presence of a quench curve.

### Immunofluorescence staining

Immunofluorescence staining was performed as described previously (Mao et al., 2021; Wu et al., 2022). Briefly, cells in a chamber slide (Thermo Fisher Scientific, #177402PK) were fixed with 4% paraformaldehyde for 10 min at room temperature and then permeabilized with 0.5% Triton X-100 solution (in phosphate-buffered saline) for 5 min. After blocking with 0.1% Triton X-100 solution (in phosphate-buffered saline) containing 5% bovine serum albumin for 1 h at room temperature, the cells were incubated with the indicated primary antibodies overnight at cold temperature. After that, the cells were washed and incubated with Alexa Fluor secondary antibodies for 1 h at room temperature. Then, cells were washed and mounted with Antifade Mounting Medium with DAPI (VECTASHIELD, #H-1200). The fluorescent images were captured using a confocal microscope (LSM 880, Zeiss).

### Real-time PCR

Real-time PCR was performed as previously described (Lee et al., 2016; Lin et al., 2014a). Briefly, total RNA was extracted using the TRIzol reagent (Life Technologies, #15596018). All cDNAs were prepared using the SuperScript II Reverse Transcriptase kit (Life Technologies, #18064-014) according to the manufacturer’s instructions. All quantitative PCRs were performed using SYBR GreenER qPCR SuperMix (Life Technologies, #11762-500). The primers for RT-PCR are listed in Table S4.

### Lipid peroxidation and cell death measurement

Lipid peroxidation was measured with BODIPY 581/591 C11 (Thermo Fisher Scientific, #D3861) as described previously (Lee et al., 2020; Lei et al., 2020). Cell death was measured using propidium iodide as described previously (Dai et al., 2017; Koppula et al., 2017). All these measurements were analyzed by fluorescence-activated cell sorting and FlowJo v10 software.

### Lipidomic analyses

#### Sample preparation

Cells were seeded in 100 mm or 150 mm culture dishes at a density of 5 × 10^6^ per dish. The next day, the cells were washed with cold PBS twice and harvested using a cell scraper. Cell pellets were collected by centrifugation at 800 ×g and snap-freeze in liquid nitrogen before storing at −80°C. Lipids were extracted from each cell pellet and analyzed as described previously (Lee et al., 2020). Briefly, samples were homogenized in ice-cold methanol containing SPLASH® LIPIDOMIX® Mass Spec Standard (Avanti Polar Lipids, Inc.) using glass bead homogenizer tubes. After homogenization, samples were transferred to fresh glass vials containing 850 μL of cold methyl-tert-butyl ether and vortex-mixed for 30 s. Next, 200 μL of ice-cold water was added, and the samples were incubated on ice for 20 min. After centrifugation (3,000 rpm for 20 min at 4°C), the lipid-containing upper phase was collected and dried down under a gentle stream of nitrogen gas. A mixture of 2-propanol/acetonitrile/water (4:3:1, *v*/*v*/*v* and 0.01% butylated hydroxytoluene) was used to reconstitute the dried samples before LC-MS analysis. A quality control sample (QC) was prepared by combining 50 μL of each sample to assess the reproducibility of the features through the runs.

#### Liquid chromatography–mass spectrometry conditions

Lipids were separated using an Acquity UPLC CSH column (2.1 × 100 mm, 1.7 μm) over a 20-min gradient elution on a Waters Acquity UPLC I-Class system. Mobile phases A—acetonitrile/water (60:40, *v*/*v*) and B—2-propanol/acetonitrile/water (85:10:5, *v*/*v*/*v*)—contained 0.1% acetic acid and 10 mmol/L ammonium acetate. Following the injections, the gradient was held at 40% mobile phase B for 2 min. At 2.1 min, it reached 50% B, then increased to 70% B in 12 min, at 12.1 min changed to 70% B, and at 18 min increased to 99% B. The eluent composition returned to the initial condition in 1 min, and the column was re-equilibrated for an additional 1 min before the next injection was conducted. The oven temperature was set at 55°C and the flow rate was 400 µL/min.

The SYNAPT G2-Si Q-ToF mass spectrometer was operated in both positive and negative electrospray ionization modes. For the positive mode, a capillary voltage and sampling cone voltage of +2 kV and 32 V were used. The source and desolvation temperatures were kept at 120°C and 500°C, respectively. Nitrogen was used as the desolvation gas with a flow rate of 800 L/h. For the negative mode, a capillary voltage of −1.5 kV and a cone voltage of 30 V was used. The source temperature was 120°C, and the desolvation gas flow was set to 800 L/h. The data were collected in duplicates in data-independent (MSE) mode over the mass range *m*/*z*: 50–1,200 Da. The quality control sample was also acquired in enhanced data-independent ion mobility (HDMSE) in both positive and negative modes for enhancing the structural assignment of lipid species. The electrospray ionization source settings for ion mobility were the same as described above. The traveling wave velocity was set to 650 m/s, and the wave height was 40 V. The helium gas flow in the helium cell region of the ion-mobility spectrometry cell was set to 180 mL/min. Nitrogen, used as the drift gas, was held at a flow rate of 95 L/min in the ion-mobility spectrometry cell. The low collision energy was set to 4 eV, and the high collision energy was ramped from 25 to 65 eV in the transfer region of the T-Wave device to induce the fragmentation of mobility-separated precursor ions.

#### Data preprocessing and analysis

All of the raw files acquired via MassLynx software (Version 4.1, waters) were imported to Progenesis QI software (Waters, Non-linear Dynamics) and aligned against the QC reference, followed by peak extraction and retention time alignment for each compound. The structural elucidation and validation of significant features were first obtained by searching monoisotopic masses against the Lipid MAPS with a mass tolerance of 5 ppm. Fragment ion information obtained by tandem MS (UPLC-HDMSE) was used for the further structural elucidation of significantly changed lipid species. HDMSE data were processed using MSE data viewer (version 1.3, Waters Corp.). Multivariate statistical analyses and the heatmap were performed using MetaboAnalyst (version 5.0) and also in an R environment. Group differences were calculated using Welch’s *t*-test. *P* values were corrected for multiple hypothesis testing, and an FDR of 0.05 or less was considered significant.

### Cell viability assay

Cell viability was measured by crystal violet staining. Briefly, cells were seeded in 96-well plates one day before treatment. The cells were washed with PBS after treatment and then stained with 0.5% crystal violet (Sigma, #C0775) dissolved in 20% methanol. After incubating for 10 min, the plates were washed with water and dried at room temperature. Then, methanol was added to solubilize the dye, and the absorbance at 540 nm was measured using a microplate reader.

### Phospholipase enzyme activity assay

The phospholipase A2 enzyme activity was measured using EnzChek™ Phospholipase A2 Assay Kit (Thermo Fisher, # E10217). Briefly, cells were lysed in NETN buffer with protease cocktail for 20 min at 4°C. The supernatant was collected after centrifugation at 16,000 ×*g* for 20 min and then was used as the substrate for PLA2 enzyme activity measurement following the instructions of the kit.

### Xenograft experiments and immunohistochemistry

The xenograft experiments were performed as previously described (Liu et al., 2023; Yan et al., 2023) and in accordance with a protocol approved by the Institutional Animal Care and Use Committee and Institutional Review Board at The University of Texas MD Anderson Cancer Center. The study is compliant with all relevant ethical regulations regarding animal research. Female athymic nude mice (Foxn1^nu^/Foxn1^nu^) of 4–6-week-old were used for cell line xenograft experiments. Mice were housed under specific-pathogen-free conditions with a 12 h light/12 h dark cycle and ambient temperature of 21–23°C with 45% humidity. HT-1080 cancer cells were resuspended in FBS-free DMEM medium and injected into mice subcutaneously. All the mice were monitored for tumor growth by bi-dimensional tumor measurements and the tumor volume was calculated according to the equation volume = 0.5 × length × width^2^. When the tumors had grown to around 50–100 mm^3^ in volume, the mice were assigned randomly into different groups (8 mice per group) and were treated with intraperitoneal injections of 30 mg/kg IKE in 100 µL of 10% dimethylsulfoxide in corn oil (vehicle), or vehicle alone daily. Animals were killed when the xenograft tumor length reached 1.5 cm.

At the end of treatment, mouse xenograft tumor samples were collected, fixed, and subjected to embedding and section. Immunohistochemical analysis was performed as previously described. Briefly, the sections after deparaffinization and rehydration were subject to antigen retrieval in the citrate-based unmasking solution (Vector Laboratories, H-3300-250) in a steam pot for 30 min. After blocking in goat serum for 1 h at room temperature, the sections were incubated with the primary antibodies anti-cleaved-caspase-3 (1:500; Cell Signaling Technology, 9661s) or anti-4-HNE (1:400; Abcam, ab46545) at 4°C overnight. The subsequent staining was performed using Vectastain elite ABC and DAB peroxidase substrate (Vector Laboratories) kits. Immunohistochemistry images were randomly taken at 400× magnification using an Olympus BX43 microscope. The immunoreactive (IRS) score was calculated according to the equation: IRS score = A (percentage of positive cells) × B (intensity of staining).

### Statistics and reproducibility

Results of cell culture experiments were obtained from at least three independent repeats. Data were represented as means ± standard deviation (SD) calculated from *n* = 3. Statistical analysis (two-tailed Student *t*-test) of bar graphs and scatter plots in this manuscript was performed using GraphPad Prism software.

## Supplementary data

The online version contains supplementary material available at https://doi.org/10.1093/procel/pwae004.

pwae004_suppl_Supplementary_Figures_S1-S8

pwae004_suppl_Supplementary_Tables_S1

pwae004_suppl_Supplementary_Tables_S2

pwae004_suppl_Supplementary_Tables_S3

pwae004_suppl_Supplementary_Tables_S4

## Data Availability

The MS proteomics data have been deposited to the ProteomeXchange Consortium via the PRIDE partner repository with the dataset identifier PXD035146. Username: reviewer_pxd034165@ebi.ac.uk; password: PqqB2OHB. All data supporting the findings of this study are available from the corresponding author on reasonable request.
