# Supplementary material for: Proteomic analysis of ferroptosis pathways reveals a role of CEPT1 in suppressing ferroptosis
Source: Protein Cell. 2024 Mar 2;15(9):686–703. doi: 10.1093/procel/pwae004 (PMC11365556; doi:10.1093/procel/pwae004)
Supplement: pwae004_suppl_Supplementary_Figures_S1-S8 [file pwae004_suppl_supplementary_figures_s1-s8.pdf]

Figure S1. Schematic of simplified ferroptosis pathways with indicated proteins used in this proteomic study. See Introduction for a detailed description.

Figure S2. Proteomic analyses of the protein-protein interaction network for ferroptosis pathways. A, Heat map showing the SAINT scores (Bayesian false discovery rate analyzed by SAINTexpress) for each of the baits and their HCIPs. **B,** Protein-protein interaction and function group of all the prey proteins that bind to the 14 ferroptosis bait proteins. **C**, Protein-protein interaction networks and function groups between 13 ferroptosis bait proteins (except LPCAT3) and their interacting proteins.

Figure S3. Schematic of phospholipid synthesis pathways. A, Schematic showing biosynthesis of phosphatidylethanolamine and phosphatidylcholine. **B,** Schematic showing the role of CEPT1 in synthesizing both phosphatidylethanolamine and phosphatidylcholine.

Figure S4. Interaction with CEPT1 prevents LPCAT3 from undergoing lysosomal proteolysis. A, Western blot analysis of indicated protein levels in 786-O and HT-1080 cells. **B,** Western blot analysis of indicated protein levels in control (sgControl) and *CEPT1*-knockout (sg1/2) HT-1080 cells. **C, D,** Statistical analysis of CEPT1 and LPCAT3 protein levels in control (sgControl) and *CEPT1*-knockout (sg1/2) HT-1080 (**C**) and 786-O (**D**) cells from three independent Western blot analyses. **E-H,** RT-PCR analyses of mRNA levels of the indicated genes in control (sgControl) and *CEPT1*-knockout (sg1/2) 786-O cells. **I-M,** RT-PCR analyses of mRNA levels of the indicated genes in control (sgControl) and *CEPT1*-knockout (sg1/2) HT-1080 cells. **N,** A pulldown assay was performed with S protein beads (SFB-tagged proteins were used as the baits), and the indicated proteins were detected by Western blot. **O,** Cystine uptake levels of control (sgControl) and *CEPT1*-knockout (sg1/2) HT-1080 cells. **P,** Western blot analysis of indicated protein levels in HT-1080 cells overexpressing CEPT1 or transfected with empty vector (EV). **Q,** RT-PCR analysis of the *LPCAT3* mRNA levels in HT-1080 cells overexpressing CEPT1 or transfected with EV. **R,** Western blot analysis of indicated protein levels in the control (Ctrl) and *CEPT1*-knockout (sg1/2) HT-1080 cells treated with DMSO or 5 μmol/L MG-132 for 24 h. **S,** Western blot analysis of indicated protein levels in the control (Ctrl) and *CEPT1*-knockout (sg1/2) 786-O cells treated with DMSO or indicated concentrations of bafilomycin A1 for 24 h. **T,** Western blot analysis of indicated protein levels in the control (Ctrl) and *CEPT1*-knockout (sg1/2) 786-O cells treated with DMSO or 40 μmol/L arachidonic acid (AA) for 24 h. **U,** Cell death was measured by PI staining in the control (sgControl) and *CEPT1*-knockout (sg1/2) 786-O cells treated with DMSO, 500 nM RSL3, 40 μmol/L arachidonic acid (AA) or indicated combination for 24 h.

Figure S5. CEPT1 inhibits ferroptosis. A, Cell death was measured by propidium iodide (PI) staining in the control (sgControl) and *LPCAT3*-knockout (sg1/2) 786-O cells treated with DMSO or 50 nM RSL3 for 24 h. **B,** Relative lipid peroxidation levels in the control (sgControl) and *CEPT1*-knockout (sg1/2) 786-O cells cultured in cystine-containing (+ Cystine) or cystine-free (- Cystine) for 8 h. **C,** Relative lipid peroxidation levels in the control (sgControl) and *CEPT1*-knockout (sg1/2) 786-O cells treated with DMSO, 10 μmol/L erastin, or 50 nM RSL3 for 8 h. **D,** Cell viability was measured in the control (sgControl) and *CEPT1*-knockout (sg1/2) HT-1080 cells treated with RSL3 for 24 h. **E,** Cell death was measured by PI staining in the control (sgControl) and *CEPT1*-knockout (sg1/2) HT-1080 cells treated with DMSO, 500 nM

RSL3, 100 μmol/L deferoxamine (DFO), or 10 μmol/L ferrostatin-1 (Fer-1) for 24 h. **F,** Cell death was measured by PI staining in the control (sgControl) and *CEPT1*-knockout (sg1/2) HT-1080 cells cultured in cystine-containing (+ Cystine) or cystine-free (- Cystine) medium with or without 100 μmol/L deferoxamine (DFO) or 10 μmol/L ferrostatin-1 (Fer-1) for 24 h. **G,** Cell death was measured by PI staining in the control (sgControl) and *CEPT1*-knockout (sg1/2) HT-1080 cells treated with DMSO, 2 μmol/L erastin, 100 μmol/L deferoxamine (DFO), or 10 μmol/L ferrostatin-1 (Fer-1) for 24 h. **H,** Cell death was measured by PI staining in HT-1080 cells overexpressing CEPT1 or transfected with EV treated with DMSO or 1 μmol/L RSL3 for 24 h. **I,** Cell death was measured by PI staining in HT-1080 cells overexpressing CEPT1 or transfected with EV cultured in cystine-containing (+ Cystine) or cystine-free (- Cystine) medium for 24 h. **J,** Cell death was measured by PI staining in the HT-1080 cells overexpressing CEPT1 or transfected with EV treated with DMSO or 5 μmol/L erastin for 24 h. **K,** Weight over time of mice with HT-1080 xenografts with the indicated treatments.

Figure S6. Loss of EPT1 or CHPT1 renders cells resistant to ferroptosis. A, Schematic showing phosphatidylethanolamine and phosphatidylcholine synthesis catalyzed by CEPT1, EPT1, and CHPT1. **B,** A pulldown assay was performed with S protein beads (SFB-tagged proteins were used as the baits), and the indicated proteins were detected by WB. **C, D,** RT-PCR analyses of *EPT1* mRNA levels (**C**) and western blotting analysis of indicated protein levels (**D**) in the control (shControl) and *EPT1*-knockdown (sh1/2) 786-O cells. **E, F,** Cell death was measured by propidium iodide (PI) staining in the control (shControl) and *EPT1*-knockdown (sh1/2) 786-O cells treated with DMSO, 50 nM RSL3, 10 μmol/L erastin, or cystine starvation (-Cystine) for 24 h. **G,** Western blot analysis of indicated protein levels in the control (sgControl) and *CHPT1*-knockout (sg1/2) 786-O cells. Vinculin was used as a loading control. **H,** Cell death was measured by PI staining in the control (sgControl) and *CHPT1*-knockout (sg1/2) 786-O cells treated with DMSO, 50 nM RSL3, 10 μmol/L erastin, or cystine starvation (- Cystine) for 24 h.

Figure S7. CEPT1 suppresses ferroptosis partly through phospholipases. A, Cell viability was measured in the control (sgCtrl) and *CEPT1-ACSL4* single or double knockout HT-1080 cells treated with RSL3 for 24 h. **B,** Schematic showing that PLA1 and PLA2 enzymes cleave fatty acyl ester bonds at the sn-1 and sn-2 sites of the glycerol backbone, respectively. **C**, **D**, Western blot analysis of indicated protein levels in the control (sgControl), *DDHD1*-knockout, or *DDHD2*-knockout (sg1/2) 786-O cells overexpressing CEPT1 or transfected with empty vector (EV). **E,** RT-PCR analysis of *ABHD3* mRNA levels in the control (shControl) and *ABHD3*-knockdown (sh1/3) 786-O cells overexpressing CEPT1 or transfected with EV. **F,** Western blot analysis of indicated protein levels in the control (sgControl) and *PLAA*-knockout (sg1/2) 786-O cells overexpressing CEPT1 or transfected with EV. **G,** An immunoprecipitation assay was performed with Myc beads to analyze interaction between Myc-tagged CEPT1 and PLAA, and the indicated proteins were detected by Western blot.

Figure S8. CEPT1 downregulates PUFA-containing phospholipids and triacylglycerols. A, Heat maps of significantly changed lipid species (One-way ANOVA; FDR-corrected p-value < 0.01; n = 4 repeats) in the control (NC) and CEPT1-knockout (KO) HT-1080 cells. **B,** Heat maps of significantly changed lipid species (One-way ANOVA; FDR-corrected p-value < 0.01; n = 4 repeats) in 786-O cells overexpressing CEPT1 WT or K138M mutant (MU) or transfected with

an empty vector (EV). Each row represents z-score-normalized intensities of the detected lipid species. Each column represents a sample. The relative abundance of each lipid is color-coded, with red indicating high signal intensity and blue indicating low signal intensity.

Fig. S1

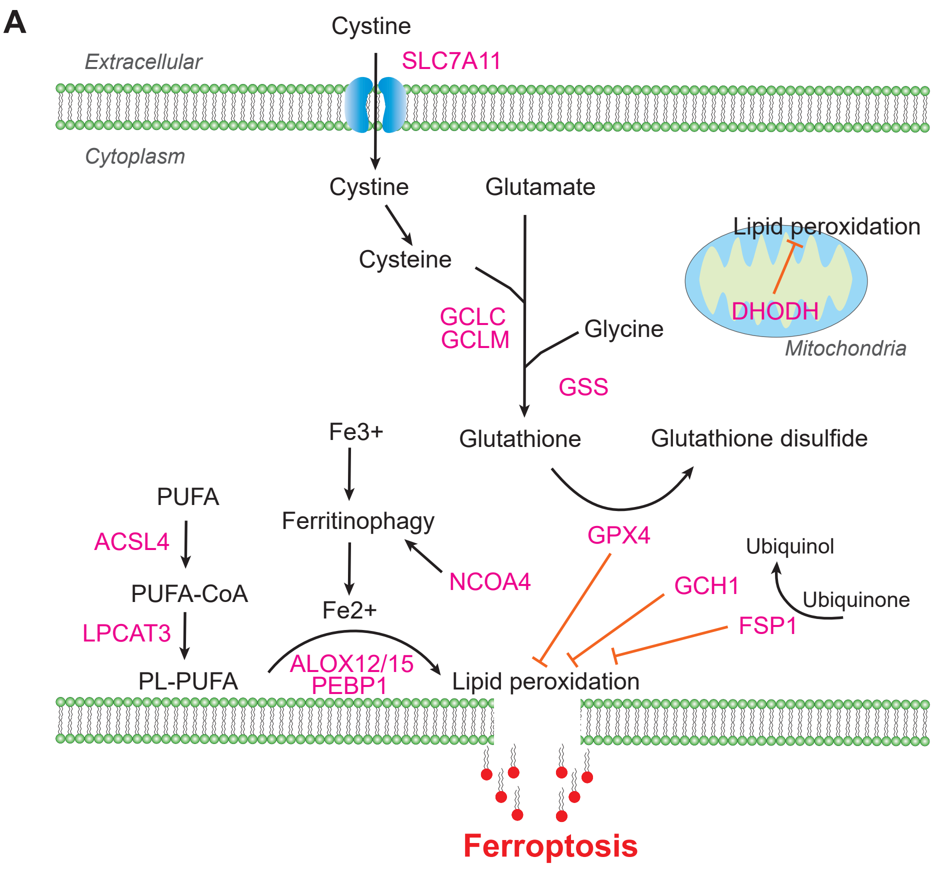
A
Cystine
SLC7A11
Extracellular
Cytoplasm
Cystine
Glutamate
Cysteine
Lipid peroxidation
DHODH
Mitochondria
GCLC
GCLM
Glycine
GSS
Fe3+
Glutathione
Glutathione disulfide
PUFA
Ferritinophagy
ACSL4
GPX4
Ubiquinol
NCOA4
PUFA-CoA
GCH1
Ubiquinone
Fe2+
LPCAT3
FSP1
ALOX12/15
PEBP1
PL-PUFA
Lipid peroxidation
Ferroptosis

Fig. S2

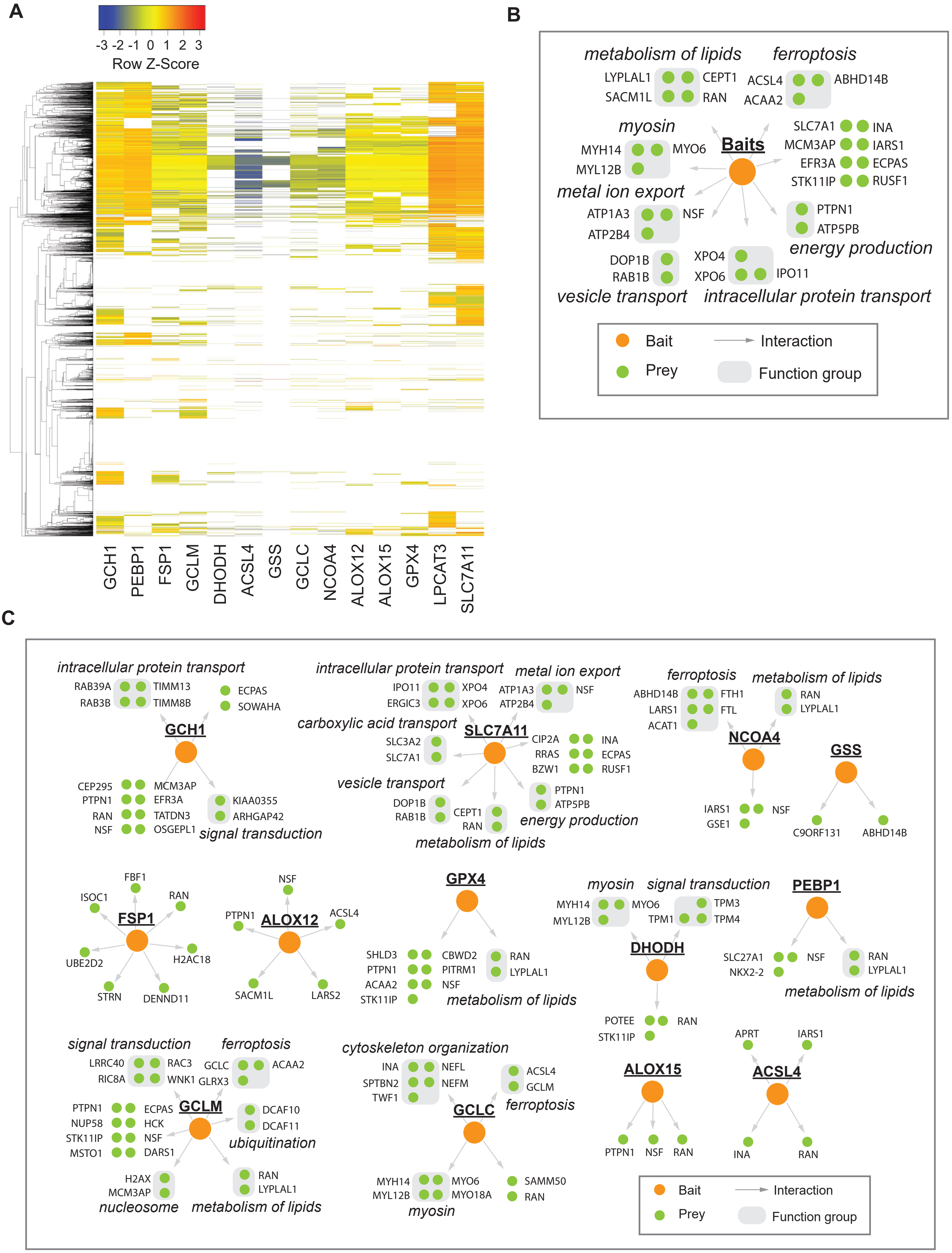
A
-3 -2 -1 0 1 2 3
Row Z-Score
GCH1
PEBP1
FSP1
GCLM
DHODH
ACSL4
GSS
GCLC
NCOA4
ALOX12
ALOX15
GPX4
LPCAT3
SLC7A11
B
metabolism of lipids
LYPLAL1
CEPT1
SACM1L
RAN
ferroptosis
ACSL4
ABHD14B
ACAA2
myosin
MYH14
MYO6
MYL12B
Baits
SLC7A1
INA
MCM3AP
IARS1
EFR3A
ECPAS
STK11IP
RUSF1
metal ion export
ATP1A3
NSF
ATP2B4
PTPN1
ATP5PB
energy production
DOP1B
RAB1B
XPO4
XPO6
IPO11
vesicle transport
intracellular protein transport
Bait
Interaction
Prey
Function group
C
intracellular protein transport
RAB39A
TIMM13
RAB3B
TIMM8B
ECPAS
SOWAHA
GCH1
CEP295
MCM3AP
PTPN1
EFR3A
RAN
TATDN3
NSF
OSGEPL1
KIAA0355
ARHGAP42
signal transduction
intracellular protein transport
IPO11
XPO4
ERGIC3
XPO6
metal ion export
ATP1A3
NSF
ATP2B4
carboxylic acid transport
SLC3A2
SLC7A1
SLC7A11
CIP2A
INA
RRAS
ECPAS
BZW1
RUSF1
vesicle transport
DOP1B
RAB1B
CEPT1
RAN
metabolism of lipids
PTPN1
ATP5PB
energy production
ferroptosis
ABHD14B
FTH1
LARS1
FTL
ACAT1
metabolism of lipids
RAN
LYPLAL1
NCOA4
IARS1
NSF
GSE1
GSS
C9ORF131
ABHD14B
FBF1
ISOC1
RAN
FSP1
UBE2D2
H2AC18
STRN
DENND11
NSF
PTPN1
ALOX12
ACSL4
SACM1L
LARS2
GPX4
SHLD3
CBWD2
PTPN1
PITRM1
ACAA2
NSF
STK11IP
RAN
LYPLAL1
metabolism of lipids
myosin
signal transduction
MYH14
MYO6
MYL12B
TPM3
TPM1
TPM4
DHODH
POTEE
RAN
STK11IP
PEBP1
SLC27A1
NSF
NKX2-2
RAN
LYPLAL1
metabolism of lipids
signal transduction
ferroptosis
LRRC40
RAC3
RIC8A
WNK1
GCLC
ACAA2
GLRX3
PTPN1
ECPAS
NUP58
HCK
STK11IP
NSF
MSTO1
DARS1
GCLM
DCAF10
DCAF11
ubiquitination
H2AX
MCM3AP
nucleosome
RAN
LYPLAL1
metabolism of lipids
cytoskeleton organization
INA
NEFL
SPTBN2
NEFM
TWF1
ACSL4
GCLM
ferroptosis
GCLC
MYH14
MYO6
MYL12B
MYO18A
myosin
SAMM50
RAN
ALOX15
PTPN1
NSF
RAN
APRT
IARS1
ACSL4
INA
RAN
Bait
Interaction
Prey
Function group

Fig. S3

A

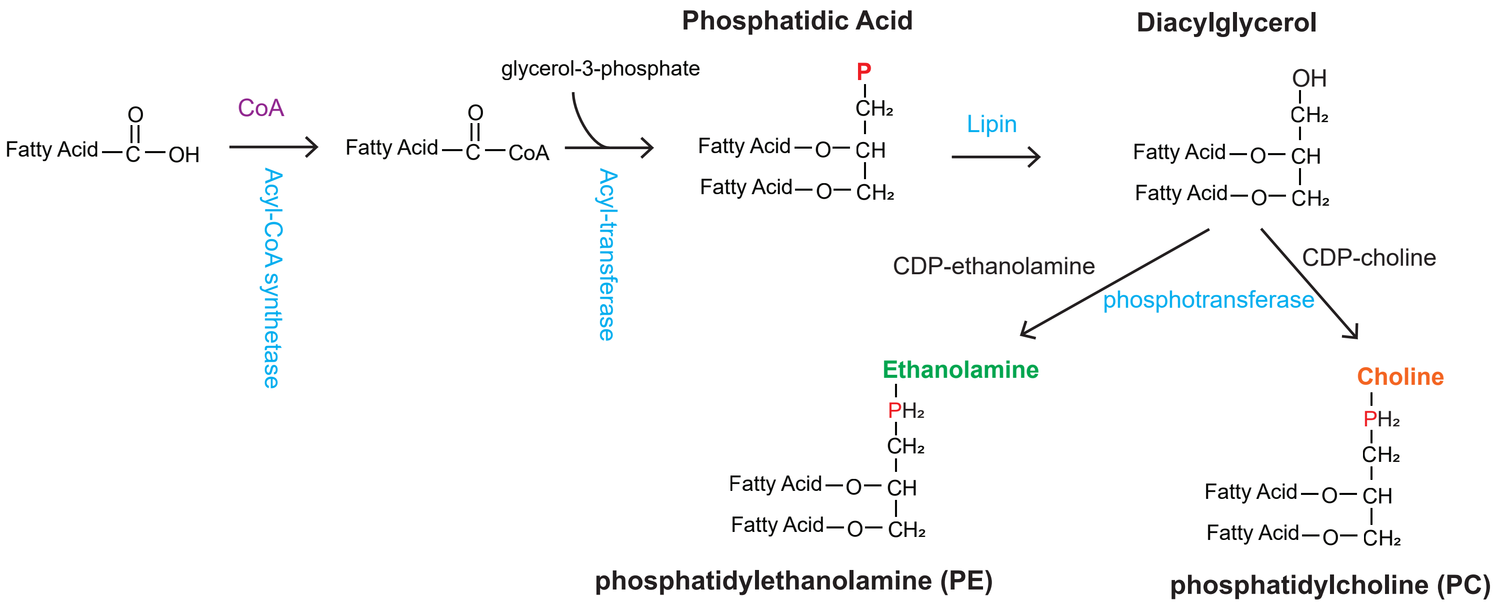
Phosphatidic Acid
Diacylglycerol
glycerol-3-phosphate
CoA
Fatty Acid—C—OH
Fatty Acid—C—CoA
Acyl-CoA synthetase
Acyl-transferase
P
CH₂
Fatty Acid—O—CH
Fatty Acid—O—CH₂
Lipin
OH
CDP-ethanolamine
CDP-choline
phosphotransferase
Ethanolamine
PH₂
Choline
phosphatidylethanolamine (PE)
phosphatidylcholine (PC)

B

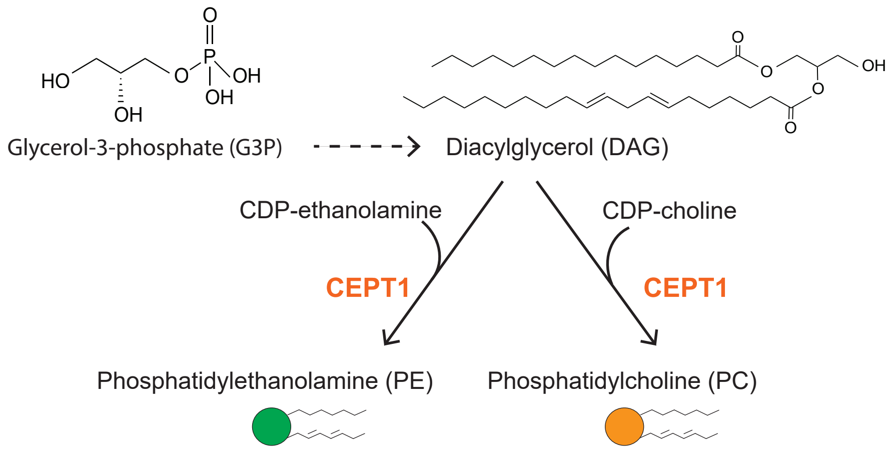
HO
OH
O
P
Glycerol-3-phosphate (G3P)
Diacylglycerol (DAG)
CDP-ethanolamine
CDP-choline
CEPT1
CEPT1
Phosphatidylethanolamine (PE)
Phosphatidylcholine (PC)

Fig. S4

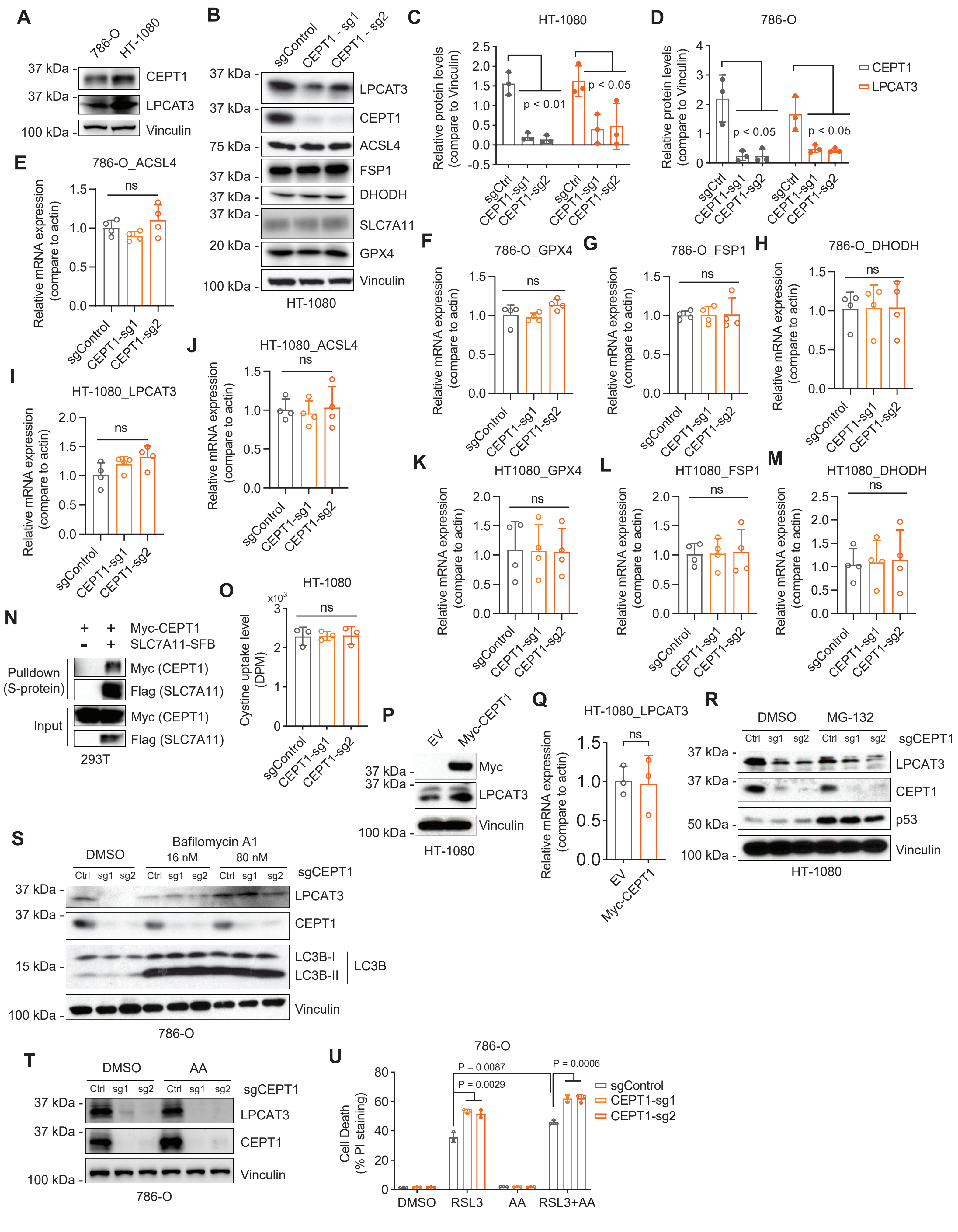

A
786-O
HT-1080
CEPT1
LPCAT3
Vinculin
B
sgControl
CEPT1 - sg1
CEPT1 - sg2
LPCAT3
CEPT1
ACSL4
FSP1
DHODH
SLC7A11
GPX4
Vinculin
HT-1080
C
HT-1080
Relative protein levels (compare to Vinculin)
p < 0.01
p < 0.05
sgCtrl
CEPT1-sg1
CEPT1-sg2
D
786-O
p < 0.05
CEPT1
LPCAT3
E
786-O_ACSL4
Relative mRNA expression (compare to actin)
ns
F
786-O_GPX4
G
786-O_FSP1
H
786-O_DHODH
I
HT-1080_LPCAT3
J
HT-1080_ACSL4
K
HT1080_GPX4
L
HT1080_FSP1
M
HT1080_DHODH
N
Myc-CEPT1
SLC7A11-SFB
Pulldown (S-protein)
Input
Myc (CEPT1)
Flag (SLC7A11)
293T
O
HT-1080
Cystine uptake level (DPM)
x10^3
P
EV
Myc-CEPT1
Myc
HT-1080
Q
HT-1080_LPCAT3
R
DMSO
MG-132
Ctrl
sg1
sg2
sgCEPT1
p53
S
Bafilomycin A1
16 nM
80 nM
LC3B-I
LC3B-II
LC3B
786-O
T
AA
U
786-O
Cell Death (% PI staining)
P = 0.0087
P = 0.0029
P = 0.0006
DMSO
RSL3
RSL3+AA
sgControl
CEPT1-sg1
CEPT1-sg2

Fig. S5

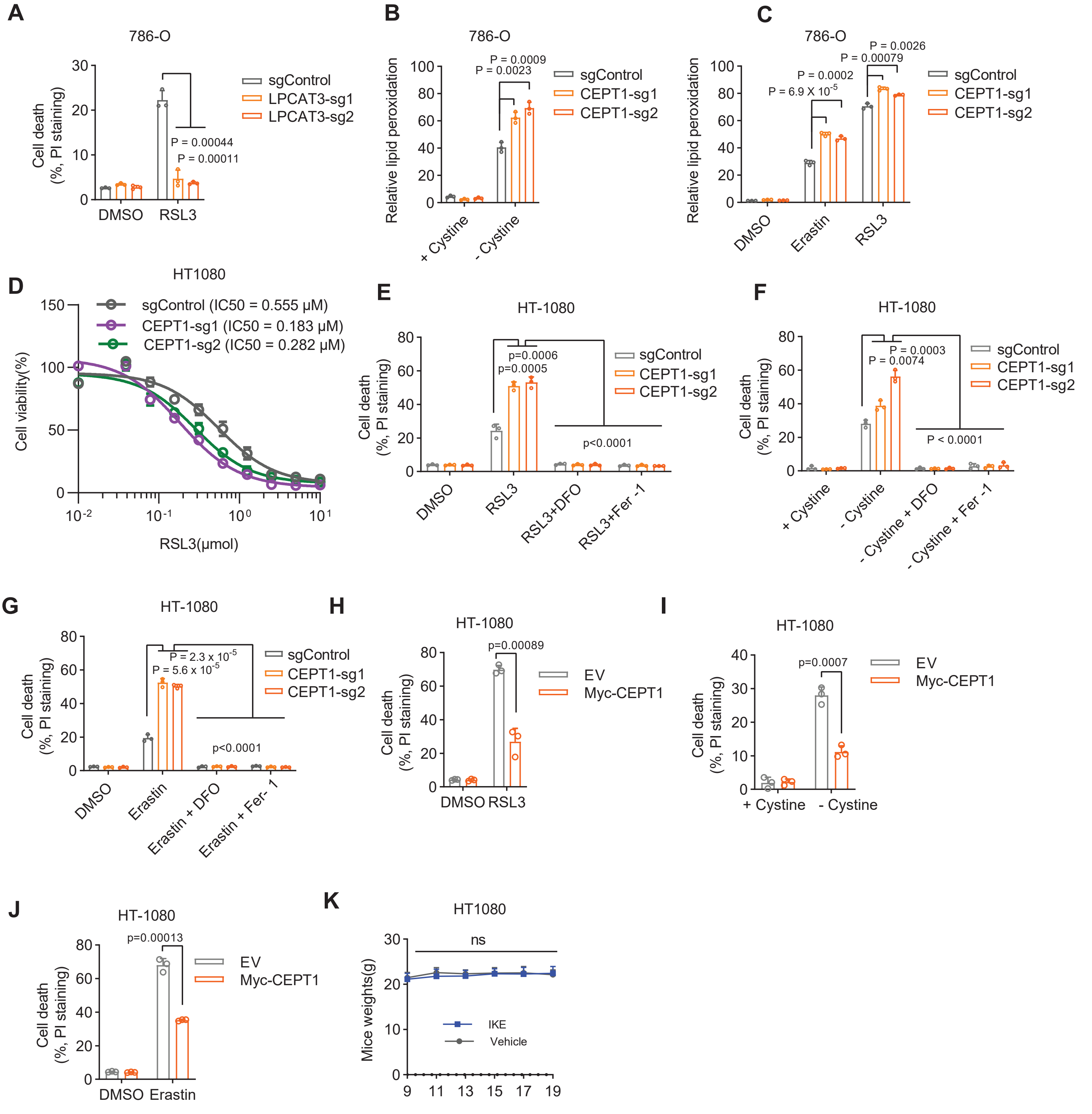

A
786-O
Cell death (%, PI staining)
sgControl
LPCAT3-sg1
LPCAT3-sg2
P = 0.00044
P = 0.00011
DMSO
RSL3
B
786-O
Relative lipid peroxidation
P = 0.0009
P = 0.0023
sgControl
CEPT1-sg1
CEPT1-sg2
+ Cystine
- Cystine
C
786-O
Relative lipid peroxidation
P = 0.0026
P = 0.00079
P = 0.0002
P = 6.9 X 10-5
sgControl
CEPT1-sg1
CEPT1-sg2
DMSO
Erastin
RSL3
D
HT1080
Cell viability(%)
sgControl (IC50 = 0.555 μM)
CEPT1-sg1 (IC50 = 0.183 μM)
CEPT1-sg2 (IC50 = 0.282 μM)
RSL3(μmol)
E
HT-1080
Cell death (%, PI staining)
p=0.0006
p=0.0005
p<0.0001
sgControl
CEPT1-sg1
CEPT1-sg2
DMSO
RSL3
RSL3+DFO
RSL3+Fer -1
F
HT-1080
Cell death (%, PI staining)
P = 0.0003
P = 0.0074
P < 0.0001
sgControl
CEPT1-sg1
CEPT1-sg2
+ Cystine
- Cystine
- Cystine + DFO
- Cystine + Fer -1
G
HT-1080
Cell death (%, PI staining)
P = 2.3 x 10-5
P = 5.6 x 10-5
p<0.0001
sgControl
CEPT1-sg1
CEPT1-sg2
DMSO
Erastin
Erastin + DFO
Erastin + Fer- 1
H
HT-1080
Cell death (%, PI staining)
p=0.00089
EV
Myc-CEPT1
DMSO
RSL3
I
HT-1080
Cell death (%, PI staining)
p=0.0007
EV
Myc-CEPT1
+ Cystine
- Cystine
J
HT-1080
Cell death (%, PI staining)
p=0.00013
EV
Myc-CEPT1
DMSO
Erastin
K
HT1080
ns
Mice weights(g)
IKE
Vehicle

Fig. S6

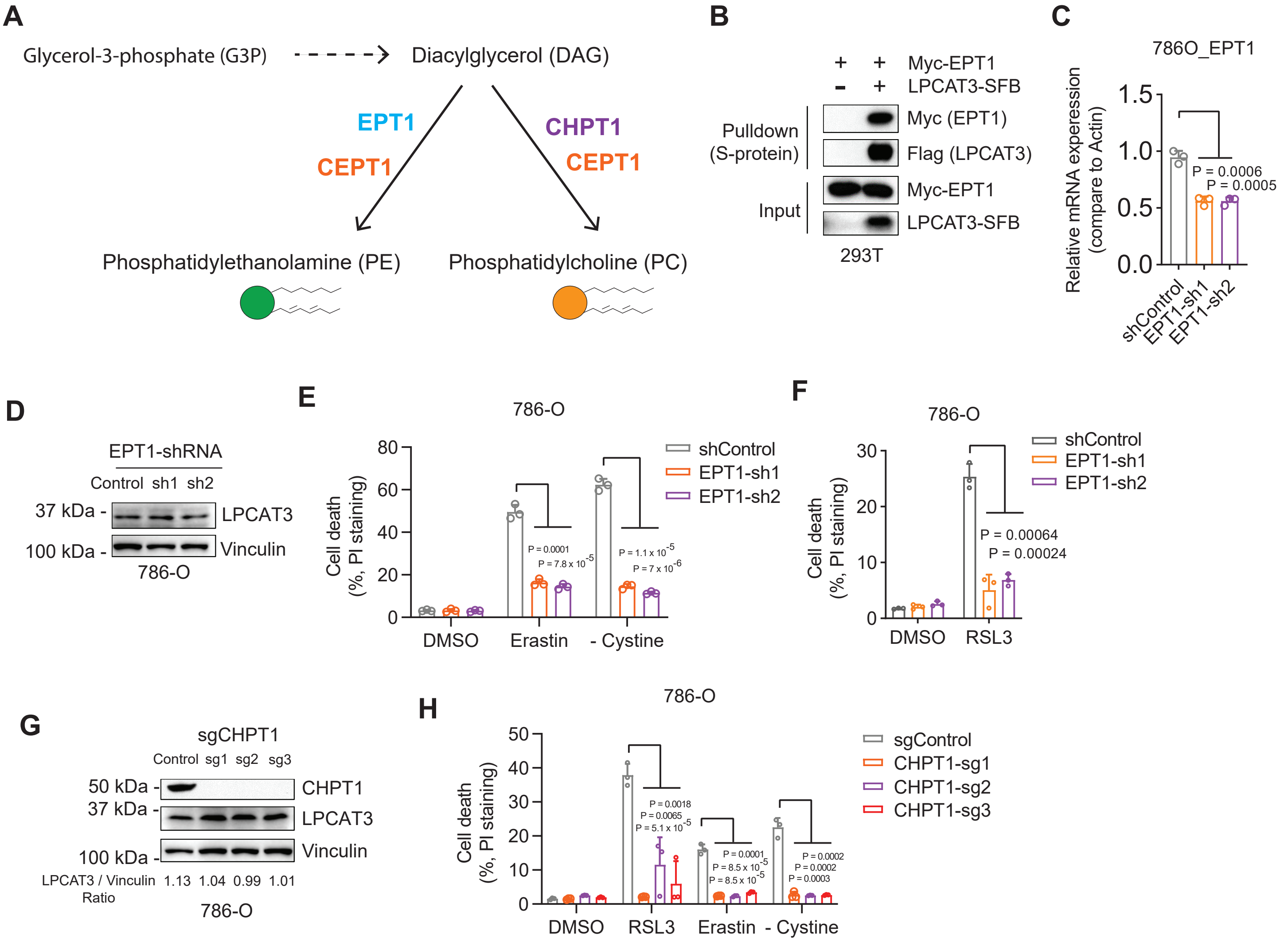

A
Glycerol-3-phosphate (G3P)
Diacylglycerol (DAG)
EPT1
CEPT1
CHPT1
CEPT1
Phosphatidylethanolamine (PE)
Phosphatidylcholine (PC)
B
Myc-EPT1
LPCAT3-SFB
Pulldown (S-protein)
Input
Myc (EPT1)
Flag (LPCAT3)
Myc-EPT1
LPCAT3-SFB
293T
C
786O_EPT1
Relative mRNA experession (compare to Actin)
P = 0.0006
P = 0.0005
shControl
EPT1-sh1
EPT1-sh2
D
EPT1-shRNA
Control sh1 sh2
37 kDa -
100 kDa -
LPCAT3
Vinculin
786-O
E
786-O
Cell death (%, PI staining)
shControl
EPT1-sh1
EPT1-sh2
DMSO
Erastin
- Cystine
F
786-O
P = 0.00064
P = 0.00024
DMSO
RSL3
G
sgCHPT1
Control sg1 sg2 sg3
50 kDa -
37 kDa -
100 kDa -
CHPT1
LPCAT3
Vinculin
LPCAT3 / Vinculin Ratio 1.13 1.04 0.99 1.01
786-O
H
786-O
sgControl
CHPT1-sg1
CHPT1-sg2
CHPT1-sg3
DMSO
RSL3
Erastin
- Cystine

Fig. S7

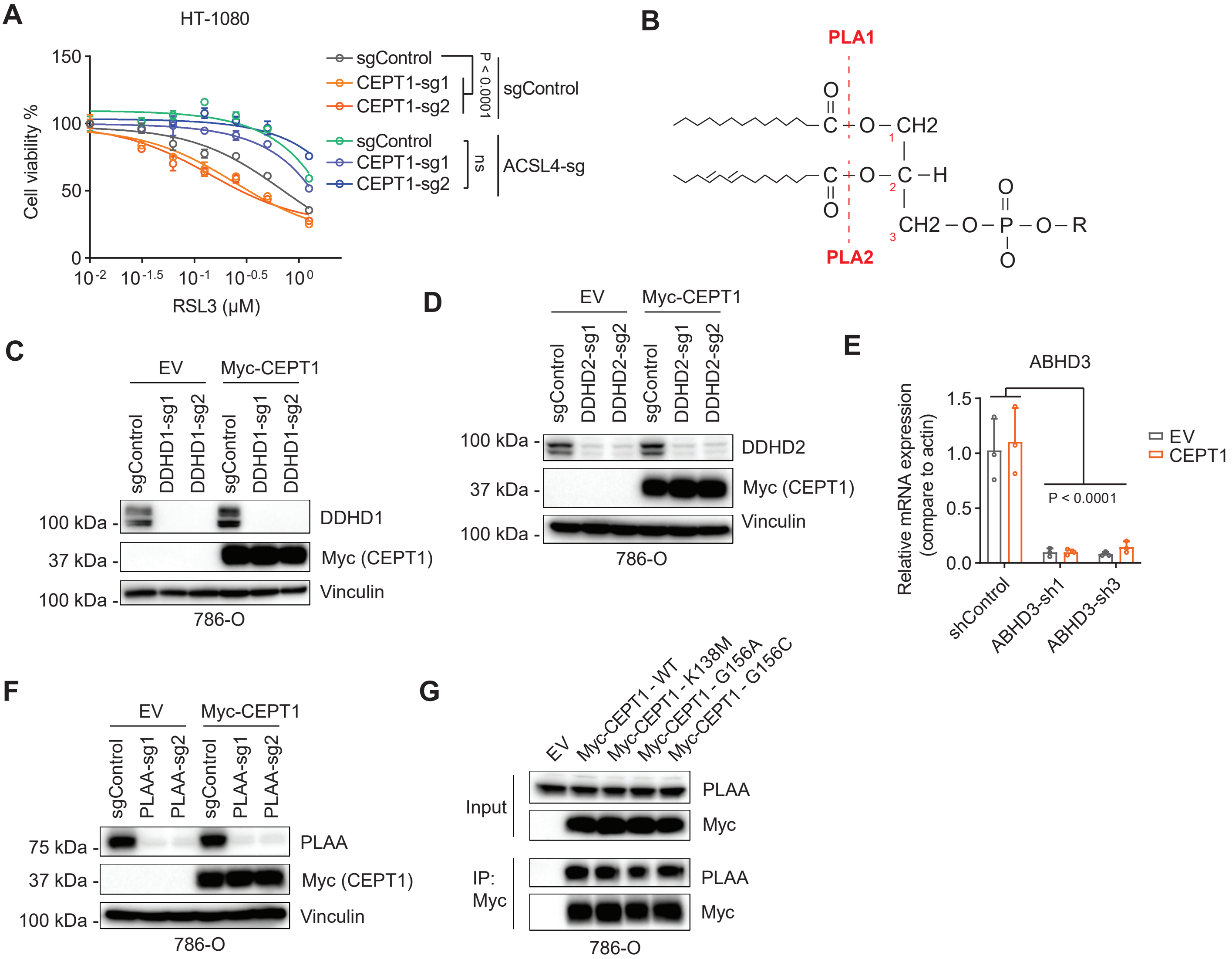
A
HT-1080
Cell viability %
RSL3 (μM)
sgControl
CEPT1-sg1
CEPT1-sg2
P < 0.0001
sgControl
ns
ACSL4-sg
B
PLA1
PLA2
C
EV
Myc-CEPT1
sgControl
DDHD1-sg1
DDHD1-sg2
100 kDa
37 kDa
DDHD1
Myc (CEPT1)
Vinculin
786-O
D
DDHD2-sg1
DDHD2-sg2
DDHD2
E
ABHD3
Relative mRNA expression (compare to actin)
EV
CEPT1
P < 0.0001
shControl
ABHD3-sh1
ABHD3-sh3
F
PLAA-sg1
PLAA-sg2
75 kDa
PLAA
G
Myc-CEPT1 - WT
Myc-CEPT1 - K138M
Myc-CEPT1 - G156A
Myc-CEPT1 - G156C
Input
IP: Myc
Myc

Fig. S8

A

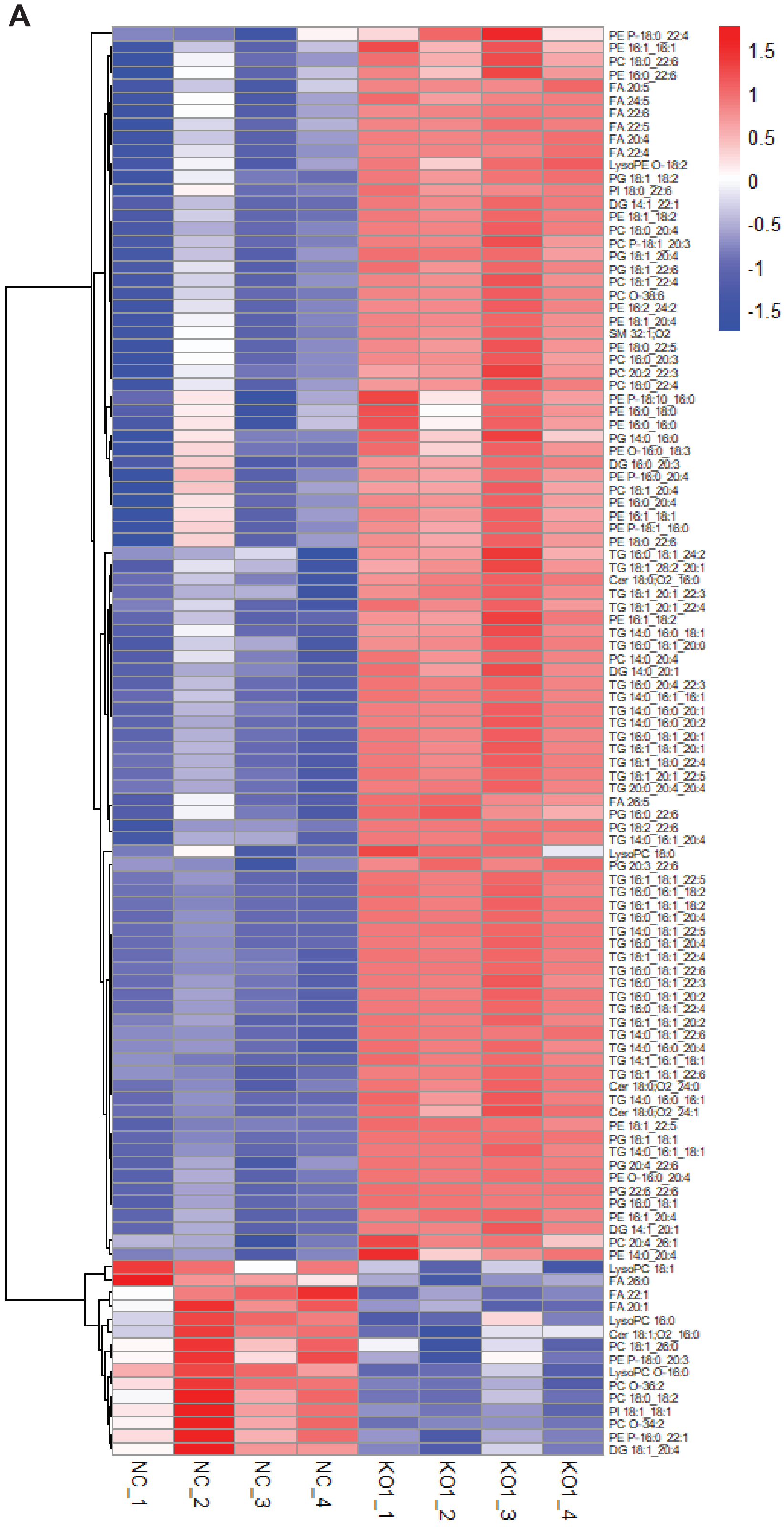
PE P-18:0_22:4
PE 16:1_18:1
PC 18:0_22:6
PE 16:0_22:6
FA 20:5
FA 24:5
FA 22:6
FA 22:5
FA 20:4
FA 22:4
LysoPE O-18:2
PG 18:1_18:2
PI 18:0_22:6
DG 14:1_22:1
PE 18:1_18:2
PC 18:0_20:4
PC P-18:1_20:3
PG 18:1_20:4
PG 18:1_22:6
PC 18:1_22:4
PC O-38:6
PE 18:2_24:2
PE 18:1_20:4
SM 32:1;O2
PE 18:0_22:5
PC 16:0_20:3
PC 20:2_22:3
PC 18:0_22:4
PE P-18:10_16:0
PE 16:0_18:0
PE 16:0_16:0
PG 14:0_16:0
PE O-16:0_18:3
DG 16:0_20:3
PE P-16:0_20:4
PC 18:1_20:4
PE 16:0_20:4
PE 16:1_18:1
PE P-18:1_16:0
PE 18:0_22:6
TG 16:0_18:1_24:2
TG 18:1_28:2_20:1
Cer 18:0;O2_16:0
TG 18:1_20:1_22:3
TG 18:1_20:1_22:4
PE 16:1_18:2
TG 14:0_16:0_18:1
TG 16:0_18:1_20:0
PC 14:0_20:4
DG 14:0_20:1
TG 16:0_20:4_22:3
TG 14:0_16:1_16:1
TG 14:0_16:0_20:1
TG 14:0_16:0_20:2
TG 16:0_18:1_20:1
TG 16:1_18:1_20:1
TG 18:1_18:0_22:4
TG 18:1_20:1_22:5
TG 20:0_20:4_20:4
FA 26:5
PG 16:0_22:6
PG 18:2_22:6
TG 14:0_16:1_20:4
LysoPC 18:0
PG 20:3_22:6
TG 16:1_18:1_22:5
TG 16:0_16:1_18:2
TG 16:1_18:1_18:2
TG 16:0_16:1_20:4
TG 14:0_18:1_22:5
TG 16:0_18:1_20:4
TG 18:1_18:1_22:4
TG 16:0_18:1_22:6
TG 16:0_18:1_22:3
TG 16:0_18:1_20:2
TG 16:0_18:1_22:4
TG 16:1_18:1_20:2
TG 14:0_18:1_22:6
TG 14:0_16:0_20:4
TG 14:1_16:1_18:1
TG 18:1_18:1_22:6
Cer 18:0;O2_24:0
TG 14:0_16:0_16:1
Cer 18:0;O2_24:1
PE 18:1_22:5
PG 18:1_18:1
TG 14:0_16:1_18:1
PG 20:4_22:6
PE O-16:0_20:4
PG 22:6_22:6
PG 16:0_18:1
PE 16:1_20:4
DG 14:1_20:1
PC 20:4_26:1
PE 14:0_20:4
LysoPC 18:1
FA 26:0
FA 22:1
FA 20:1
LysoPC 16:0
Cer 18:1;O2_16:0
PC 18:1_26:0
PE P-18:0_20:3
LysoPC O-16:0
PC O-36:2
PC 18:0_18:2
PI 18:1_18:1
PC O-34:2
PE P-16:0_22:1
DG 18:1_20:4
1.5
1
0.5
0
-0.5
-1
-1.5
NC_1
NC_2
NC_3
NC_4
KO1_1
KO1_2
KO1_3
KO1_4

B

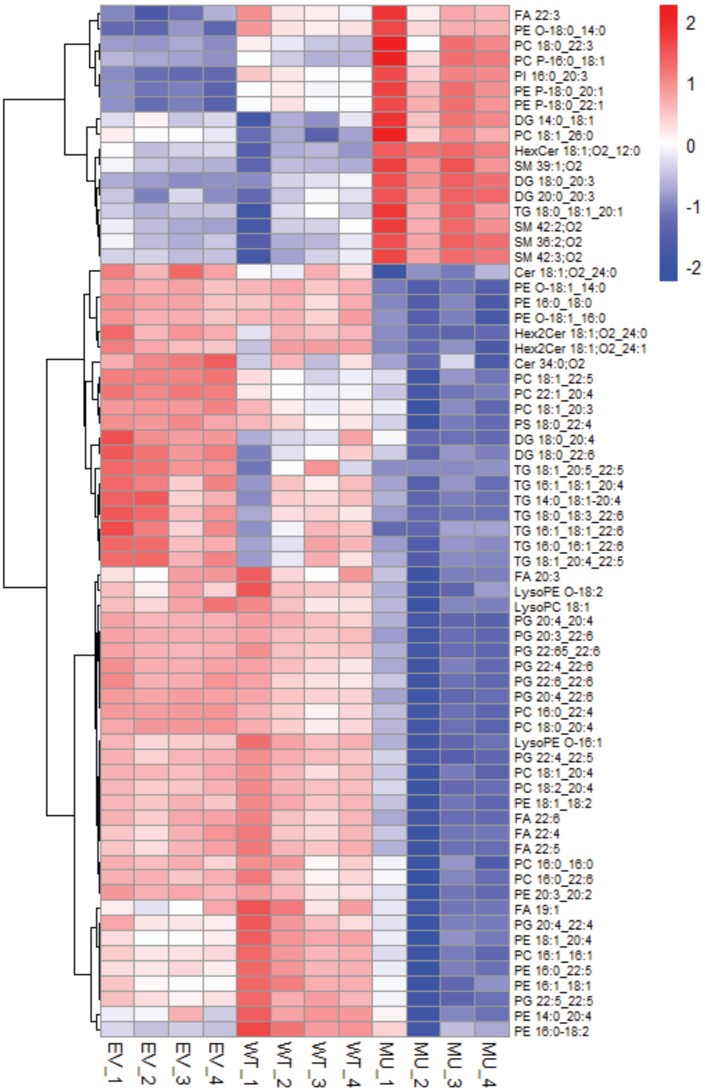
FA 22:3
PE O-18:0_14:0
PC 18:0_22:3
PC P-16:0_18:1
PI 16:0_20:3
PE P-18:0_20:1
PE P-18:0_22:1
DG 14:0_18:1
PC 18:1_26:0
HexCer 18:1;O2_12:0
SM 39:1;O2
DG 18:0_20:3
DG 20:0_20:3
TG 18:0_18:1_20:1
SM 42:2;O2
SM 36:2;O2
SM 42:3;O2
Cer 18:1;O2_24:0
PE O-18:1_14:0
PE 16:0_18:0
PE O-18:1_16:0
Hex2Cer 18:1;O2_24:0
Hex2Cer 18:1;O2_24:1
Cer 34:0;O2
PC 18:1_22:5
PC 22:1_20:4
PC 18:1_20:3
PS 18:0_22:4
DG 18:0_20:4
DG 18:0_22:6
TG 18:1_20:5_22:5
TG 16:1_18:1_20:4
TG 14:0_18:1-20:4
TG 18:0_18:3_22:6
TG 16:1_18:1_22:6
TG 16:0_16:1_22:6
TG 18:1_20:4_22:5
FA 20:3
LysoPE O-18:2
LysoPC 18:1
PG 20:4_20:4
PG 20:3_22:6
PG 22:65_22:6
PG 22:4_22:6
PG 22:6_22:6
PG 20:4_22:6
PC 16:0_22:4
PC 18:0_20:4
LysoPE O-16:1
PG 22:4_22:5
PC 18:1_20:4
PC 18:2_20:4
PE 18:1_18:2
FA 22:6
FA 22:4
FA 22:5
PC 16:0_16:0
PC 16:0_22:6
PE 20:3_20:2
FA 19:1
PG 20:4_22:4
PE 18:1_20:4
PC 16:1_16:1
PE 16:0_22:5
PE 16:1_18:1
PG 22:5_22:5
PE 14:0_20:4
PE 16:0-18:2
2
1
0
-1
-2
EV_1
EV_2
EV_3
EV_4
WT_1
WT_2
WT_3
WT_4
MU_1
MU_2
MU_3
MU_4
